# ﻿Survey of the Notonectidae (Insecta, Hemiptera, Heteroptera, Nepomorpha) from northeastern Brazil

**DOI:** 10.3897/zookeys.1233.120598

**Published:** 2025-04-01

**Authors:** Rafael Jordão, Julianna Freires Barbosa, Felipe Ferraz Figueiredo Moreira

**Affiliations:** 1 Laboratório de Biodiversidade Entomológica, Instituto Oswaldo Cruz, Fundação Oswaldo Cruz, Rio de Janeiro, Brazil Instituto Oswaldo Cruz, Fundação Oswaldo Cruz Rio de Janeiro Brazil; 2 Laboratório de Entomologia, Departamento de Zoologia, Instituto de Biologia, Universidade Federal do Rio de Janeiro, Rio de Janeiro, Brazil Universidade Federal do Rio de Janeiro Rio de Janeiro Brazil

**Keywords:** Aquatic insects, Atlantic Forest, backswimmers, biodiversity, Caatinga

## Abstract

A four-year survey for Notonectidae (Insecta: Hemiptera: Heteroptera: Nepomorpha) was conducted in eleven conservation units and adjacent areas distributed over six states in northeastern Brazil. Nearly 1400 specimens of the following 18 species, four genera, and two subfamilies have been collected: *Buenoaamnigenus* (White, 1879), *B.femoralis* (Fieber, 1851), *B.fuscipennis* (Berg, 1879), *B.koina* Nieser & Pelli, 1994, *B.konta* Nieser & Pelli, 1994, *B.mutabilis* Truxal, 1953, *B.pallipes* (Fabricius, 1803), *B.platycnemis* (Fieber, 1851), *B.pseudomutabilis* Barbosa, Ribeiro & Nessimian, 2010, *B.salutis* Kirkaldy, 1904, *B.tarsalis* Truxal, 1953, *B.unguis* Truxal, 1953 (Anisopinae); *Enitharoidesbrasiliensis* (Spinola, 1837), *E.tricomerus* Barbosa, Ribeiro & Nessimian, 2017, *Martaregabentoi* Truxal, 1949, *M.brasiliensis* Truxal, 1949, *M.membranacea* White, 1879, and *Notonectadisturbata* Hungerford, 1926 (Notonectinae). Altogether, they represent 30 new records from the states of Alagoas, Bahia, Ceará, Pernambuco, Piauí, and Sergipe, and seven new records from northeastern Brazil, increasing the number of species recorded from the region from 11 to 18. A key to these species is provided, as well as illustrations, diagnoses, taxonomic notes, and a summary of their known geographic distributions.

## ﻿Introduction

Notonectidae (Insecta: Hemiptera: Heteroptera: Nepomorpha) is a cosmopolitan family and second in richness only to Corixidae sensu lato among the aquatic bugs, with approximately 400 species and 11 genera (Polhemus and Polhemus 2008). Commonly known as backswimmers, because of the remarkable upside-down swimming method of all species in the family, they exhibit fusiform body up to 15 mm in length (Schuh and Slater 1995). They can be found in lotic and lentic habitats, like slow streams, artificial and natural ponds, and even small mud puddles, feeding on aquatic organisms such as mosquito larvae, crustaceans, and fish larvae (Papáček 2000). Notonectids go through four or five nymphal stages during their development and show wing polymorphism in some genera (Hungerford 1933). The family is currently divided in two subfamilies, Anisopinae and Notonectinae, both of which are present in Brazil. The former is represented in the country by 31 species of the genus *Buenoa* Kirkaldy, 1904, and the latter by *Enitharoides* Brooks, 1953 (four species), *Notonecta* Fabricius, 1758 (eight species), and *Martarega* White, 1879 (fourteen species) (Moreira et al. 2011; Ribeiro et al. 2022).

Most of the data concerning backswimmers from Brazil are based on material collected in its southeastern portion (Moreira et al. 2011), with some recent advances in the northern, northeastern, and central-western regions (e.g., Barbosa et al. 2010a, b, 2012; Barbosa and Nessimian 2013a, b; Barbosa and Giehl 2014; Moreira et al. 2016; Takiya et al. 2016; Barbosa and Dias-Silva 2017). The Caatinga biome spreads through nearly 11% of the Brazilian territory, contained within nine states of the northeastern region and a small portion of northern Minas Gerais state (southeastern region). Surrounded by the Amazon, *Cerrado*, and Atlantic Forest biomes, it is a semiarid and dynamic environment.

The biodiversity of the Caatinga currently faces two large threats, namely the deforestation caused by cattle herding and climate change, both of which can interfere with the already erratic local rainfall regime and lead to a desertification process (Castelletti et al. 2004; Oyama and Nobre 2004). Moreover, recent observations indicate that conservation units in Brazil fail to effectively safeguard aquatic insects, as the protection they afford is comparable to that of randomly chosen areas. A primary contributing factor to this inefficiency is the haphazard selection of sites for development, often motivated by economic considerations or anecdotal evidence (Dias-Silva et al. 2021). Considering this scenario, we present here the partial results of a survey of the Nepomorpha from northeastern Brazil, focusing on the family Notonectidae and mainly on the Caatinga biome, but also including adjacent areas of Atlantic Forest.

## ﻿Materials and methods

Specimens collected in Alagoas (AL) and Sergipe (SE) states were obtained in 2018 and 2019 as part of the project “Diversity and distribution of water bugs (Insecta: Heteroptera: Gerromorpha and Nepomorpha) of Alagoas and Sergipe, northeastern Brazil”. Specimens collected in Bahia (BA), Ceará (CE), Pernambuco, (PE), and Piauí (PI) states are results of the project “Diversity and conservation of Hemiptera (Insecta) from the Caatinga”, carried out from 2018 to 2021. The following eleven federal conservation units have been studied (Figs 11–20): Área de Proteção Ambiental da Costa dos Corais (APACC, AL) , Área de Proteção Ambiental de Piaçabuçu (APAP, AL) , Estação Ecológica de Murici (EEM, AL) , Reserva Extrativista Marinha da Lagoa do Jequiá (RESEX, AL) , Reserva Biológica de Pedra Talhada (RBPT, AL/PE) , Parque Nacional da Chapada Diamantina (PNCD, BA) , Estação Ecológica de Aiuaba (EEA, CE) , Parque Nacional do Catimbau (PNCA, PE) , Parque Nacional da Serra das Confusões (PNSC, PI) , Parque Nacional da Serra de Itabaiana (PNSI, SE) , and Reserva Biológica de Santa Isabel (RBSI, SE). Brazilian states are abbreviated as follows: Acre (AC) ; Alagoas (AL) ; Amapá (AP) ; Amazonas (AM) ; Bahia (BA) ; Ceará (CE) ; Distrito Federal (DF) ; Espírito Santo (ES) ; Goiás (GO) ; Maranhão (MA) ; Mato Grosso (MT) ; Mato Grosso do Sul (MS) ; Minas Gerais (MG) ; Pará (PA) ; Paraíba (PB) ; Paraná (PR) ; Pernambuco (PE) ; Piauí (PI) ; Rio de Janeiro (RJ) ; Rio Grande do Norte (RN) ; Rio Grande do Sul (RS) ; Rondônia (RO) ; Roraima (RR) ; Santa Catarina (SC) ; São Paulo (SP) ; Sergipe (SE) ; Tocantins (TO).

The collecting methods employed were light traps (white sheet) and active sampling with sieves and aquatic nets in water bodies like swamps, puddles, streams, lakes, and small rivers. Specimen identification was based mostly on Truxal (1953), Barbosa and Nessimian (2013a, b), and Barbosa et al. (2017). Images of the specimens were acquired with a Leica M205C stereomicroscope at different focal distances and stacked using LAS CORE v. 4.6. Image enhancements were made using Adobe Photoshop CC 2015. Drawings and photographs were made based on male specimens. Material examined is deposited in the Coleção Entomológica do Instituto Oswaldo Cruz, Rio de Janeiro, Brazil (**CEIOC**), and in the Coleção Zoológica do Maranhão, Caxias, Brazil (CZMA).

In the distribution section of each species, all references that we are aware of are provided for the records from Brazilian states. For other countries or territories, only the first known record is provided. New state records are preceded by an exclamation mark “!” in the lists of material examined. Distribution maps were made with QGIS v. 3.16.3 (QGIS 2022). Truxal (1953) recorded *Buenoaantigoneantigone* (Kirkaldy, 1899) from northeastern Brazil based on female specimens only, which is questionable. Because this is the only record of this species from the study area and it is absent from our samples, we decided to not include it in our results.

## ﻿Results

### ﻿Key to males of Notonectidae species found in northeastern Brazil

**Table:** 

1	Labrum triangular (Fig. 1C, D); hemelytral commissure without hair-lined pit (Fig. 1A)	[Notonectinae] **2**
–	Labrum rounded (Fig. 1E); hemelytral commissure with hair-lined pit (Fig. 1B)	[Anisopinae, *Buenoa*] **7**
2	Middle femur with anteapical pointed spine (Fig. 2C, F)	[Notonectini] **3**
–	Middle femur without such structure	[Nychiini, *Martarega*] **5**
3	Anterolateral margins of pronotum straight, without foveae (Fig. 2D); male parameres symmetrical	***Notonectadisturbata* Hungerford, 1926** (Fig. 10M)
–	Anterolateral margins of pronotum foveate (Fig. 2A); male parameres asymmetrical	[*Enitharoides*] **4**
4	Scutellum with wide, subrectangular, punctate area medially (Fig. 2B); middle femur with short pubescence and poorly developed anteapical protuberance (Fig. 2C)	***Enitharoidesbrasiliensis* (Spinola, 1837)** (Fig. 10N)
–	Scutellum with widely distributed, deeply punctate area (Fig. 2E); middle femur with long pubescence and robust anteapical protuberance (Fig. 2F)	***Enitharoidestricomerus* Barbosa, Ribeiro & Nessimian, 2017** (Fig. 10O)
5	Ocular commissure < 1/2 of eye width (Fig. 6A); ventral surface of middle trochanter with a small group of ensiform bristles centrally (Fig. 3A)	***Martaregabentoi* Truxal, 1949** (Fig. 10P, Q)
–	Ocular commissure 1/2 of eye width or more; ventral surface of middle trochanter with a patch of thin setae	**6**
6	Ocular commissure half of eye width; third labial article pubescent; scutellum with hyaline apex (Fig. 3B)	***Martaregabrasiliensis* Truxal, 1949** (Fig. 10R)
–	Ocular commissure more than half of eye width; third labial article glabrous; scutellum with posterior half of its length hyaline (Fig. 3C)	***Martaregamembranacea* White, 1879** (Fig. 10S)
7	Synthlipsis wide, half of anterior width of vertex or slightly less (Fig. 6B)	**8**
–	Synthlipsis narrow, less than half of anterior width of vertex	**12**
8	Labial prong longer than third labial article (Fig. 6C)	**9**
–	Labial prong equal to or shorter than third labial article	**11**
9	Apex of fore femur narrowed (length > 3× the width at apex, Fig. 6D); middle tarsus with first article deeply emarginated (Fig. 4A)	***Buenoatarsalis* Truxal, 1953** (Fig. 10K)
–	Apex of fore femur slightly widened (length ≤ 3× width at apex); middle tarsus without such modification	**10**
10	Large species (7.8–8.6 mm); labial prong slightly longer than third labial article, nearly straight (Fig. 7A)	***Buenoafemoralis* (Fieber, 1851)** (Fig. 10B)
–	Medium-sized species (5.5–6.2 mm); labial prong much longer than third labial article, arc-shaped (Fig. 6C)	***Buenoapallipes* (Fabricius, 1803)** (Fig. 10G)
11	Labial prong subequal to slightly longer than third labial article length; fore trochanter without conspicuous process pointing backwards; fore femur widened at apex; middle tibia not widened at apex	***Buenoakoina* Nieser & Pelli, 1994** (Fig. 10D)
–	Labial prong distinctly shorter than third labial article (Fig. 7D); fore trochanter with a conspicuous process pointing backwards (Fig. 4B); fore femur not widened at apex; middle tibia widened at apex	***Buenoafuscipennis* (Berg, 1879)** (Fig. 10C)
12	Fore femur with stridulatory area (Fig. 6D, small arrow)	**13**
–	Fore femur without stridulatory area	**15**
13	Labial prong originating distally on third labial article; apex of fore femur widened	***Buenoaplatycnemis* (Fieber, 1851)** (Fig. 10H)
–	Labial prong originating proximally or medially on third labial article; apex of fore femur narrowed	**14**
14	Tylus pubescent; stridulatory area of foretibia with 21–25 teeth; abdominal ventral laterotergite I with a dark nodule (Fig. 5B)	***Buenoapseudomutabilis* Barbosa, Ribeiro & Nessimian, 2010** (Fig. 10I)
–	Tylus glabrous; stridulatory area of foretibia with 33–38 teeth; abdominal ventral laterotergite I without dark nodule	***Buenoamutabilis* Truxal, 1953** (Fig. 10F)
15	Tylus flat; labial prong shorter than third labial article; fore tarsus with claws orthogonal-angulated (Fig. 5A)	***Buenoaunguis* Truxal, 1953** (Fig. 10L)
–	Tylus rounded; labial prong subequal to or longer than third labial article; fore tarsus with unmodified claws	**16**
16	Synthilipsis ~ 1/4 of vertex width; blackish brown hoof print-like pattern at hemelytra in dorsal view; apex of fore femur widened	***Buenoakonta* Nieser & Pelli, 1994** (Fig. 10E)
–	Synthilipsis < 1/4 of vertex width; hemelytra without such pattern; apex of fore femur not widened	**17**
17	Synthlipsis very narrow, < 1/10 of vertex width; frons narrow; labrum sided by pair of tufts of setae (Fig. 7B); labial prong slightly longer than third labial article, with apex rounded	***Buenoaamnigenus* (White, 1879)** (Fig. 10A)
–	Synthlipsis narrow, > 1/10 of vertex width; frons wide; labrum without tufts of setae; labial prong subequal to slightly longer than third labial article, with apex sinuous (Fig. 7C)	***Buenoasalutis* Kirkaldy, 1904** (Fig. 10J).

**Figure 1.**
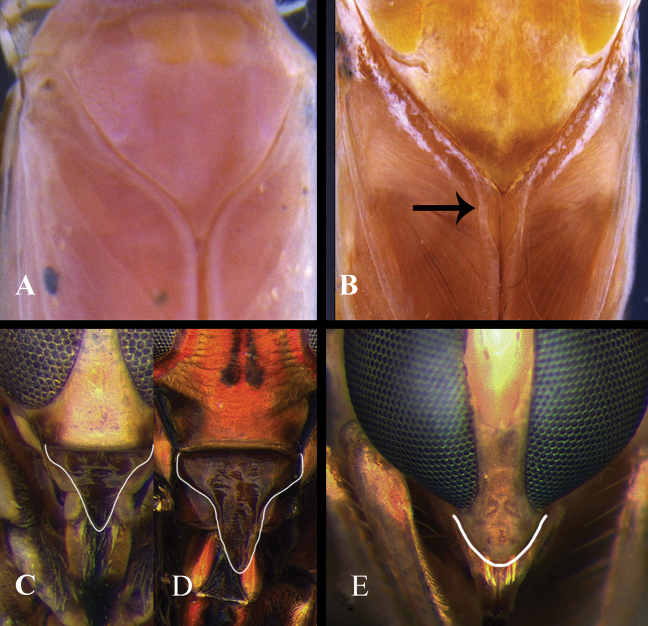
Anisopinae and Notonectinae, examples of structures **A, B** scutellum and anterior part of hemelytra, dorsal view **A**Notonectinae**B**Anisopinae**C–E** head, anterior view, outline of labrum highlighted **C***Martarega***D***Enitharoides***E***Buenoa*.

**Figure 2.**
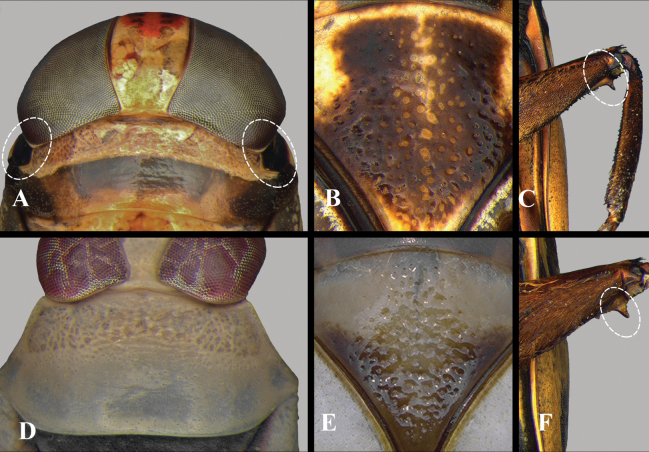
Notonectini, morphological characteristics **A***Enitharoides*, foveate anterolateral portion of pronotum delimited by dotted line **B, C***Enitharoidesbrasiliensis***B** punctation of pronotum, dorsal view **C** middle femur, ventral view **D***Notonectadisturbata*, unfoveate anterolateral portion of pronotum **E, F***Enitharoidestricomerus***E** punctation of pronotum, dorsal view **F** middle femur, ventral view.

**Figure 3.**
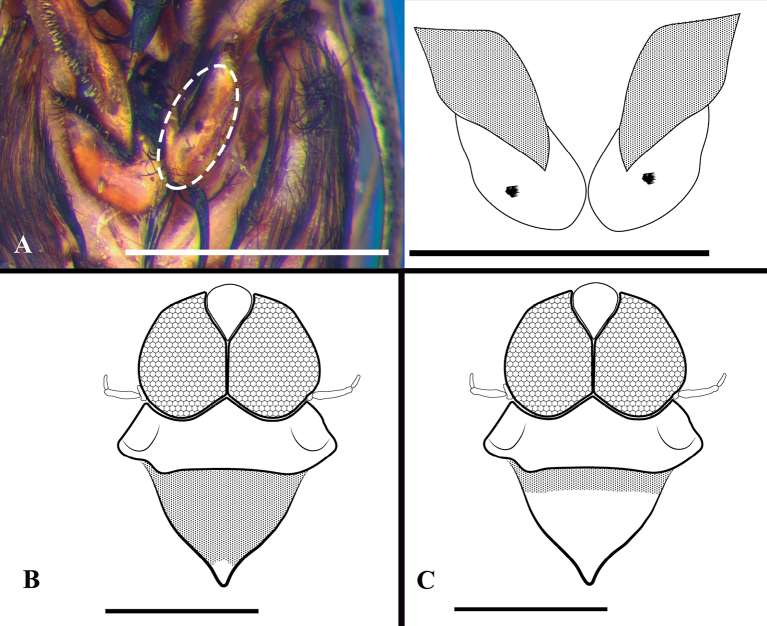
*Martarega*, morphological characteristics **A***Martaregabentoi*, middle trochanters, ventral view, small groups of ensiform bristles delimited by dotted line. Trochanters of right image without dots **B***M.brasiliensis*, dorsal view of scutellum, hyaline apex delimited by absence of dotted pattern **C***M.membranacea*, dorsal view of scutellum.

**Figure 4.**
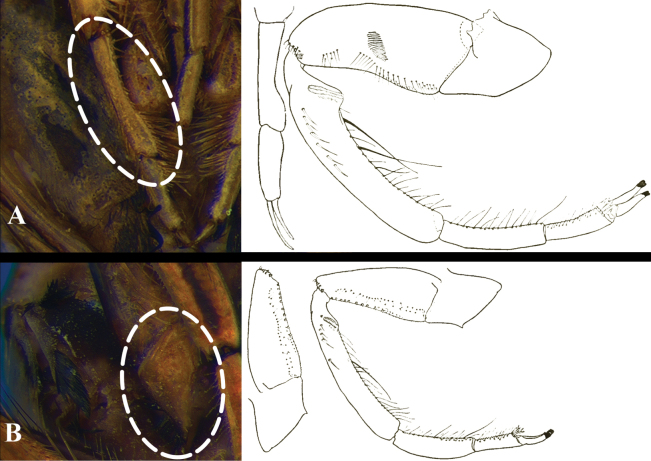
*Buenoa*, structures of two species, highlighted with black dots in right illustrations **A***B.tarsalis*, emarginated first article of middle tarsus, ventral view **B***B.fuscipennis*, posteroventral portion of fore trochanters, ventral view. Right illustrations modified from Truxal 1953.

**Figure 5.**
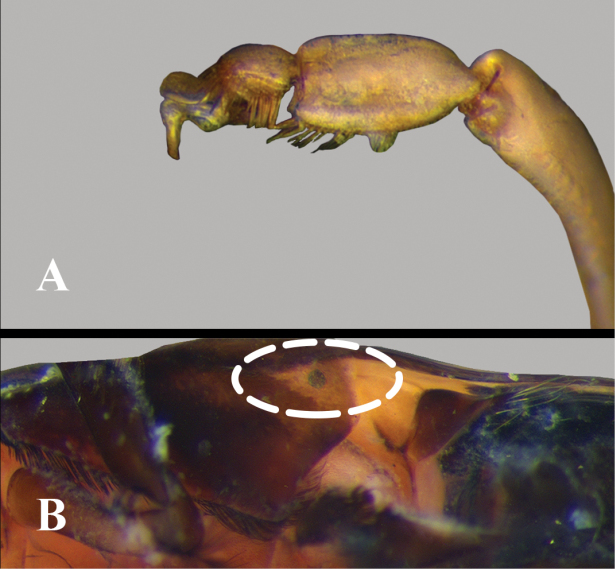
*Buenoa*, structures of two species **A***B.unguis*, male fore tarsus, ventral view **B***B.pseudomutabilis*, male abdominal ventral laterotergite I, ventral view.

**Figure 6.**
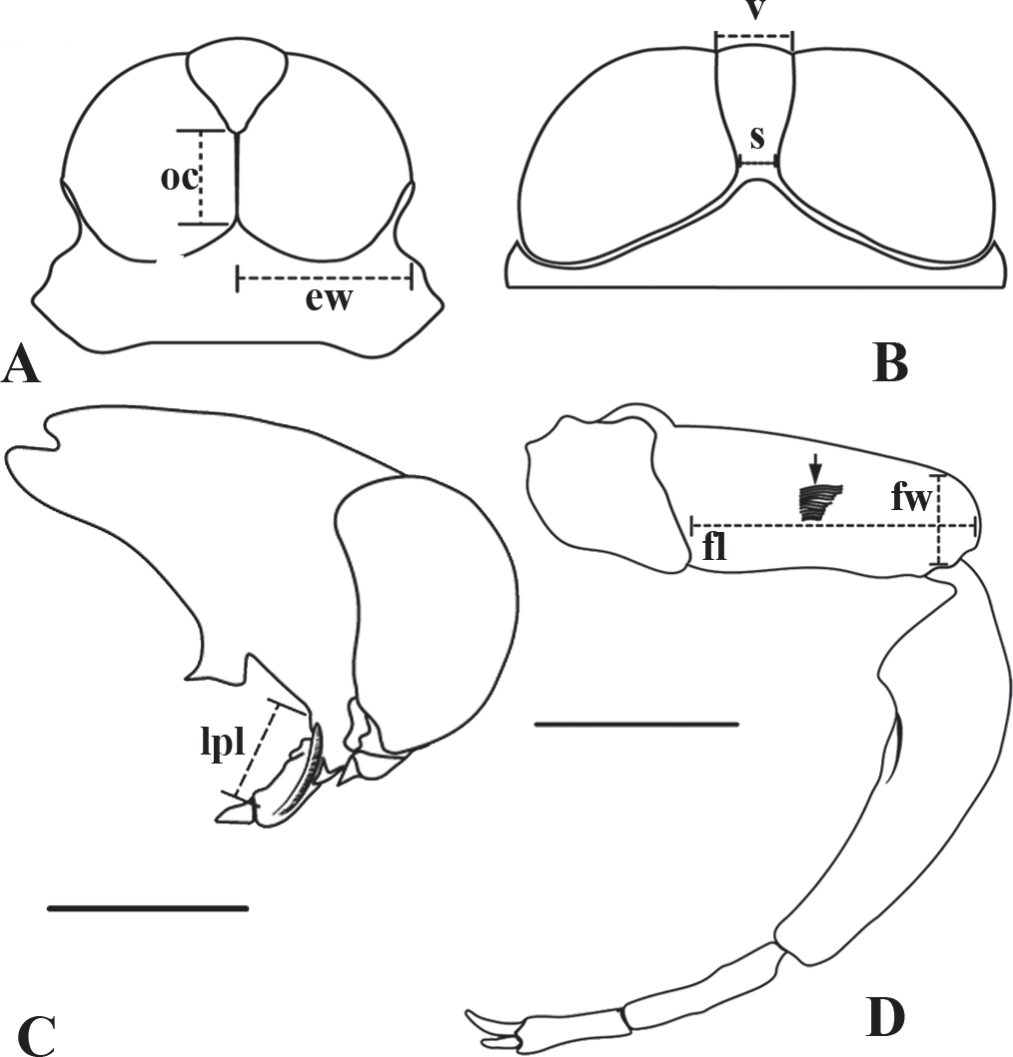
*Martarega* and *Buenoa*, measurements of head and fore femur **A***Martarega*, head, dorsal view **B–D***Buenoa***B** head, dorsal view **C** length of labial prong, lateral view **D** fore femur, ventral view, black arrow indicates stridulatory area. Abbreviations: ew = eye width, fl = femoral length, fw = femoral width, oc = ocular commissure, s = synthlipsis width, v = vertex width. Scale bars: 1 mm.

**Figure 7.**
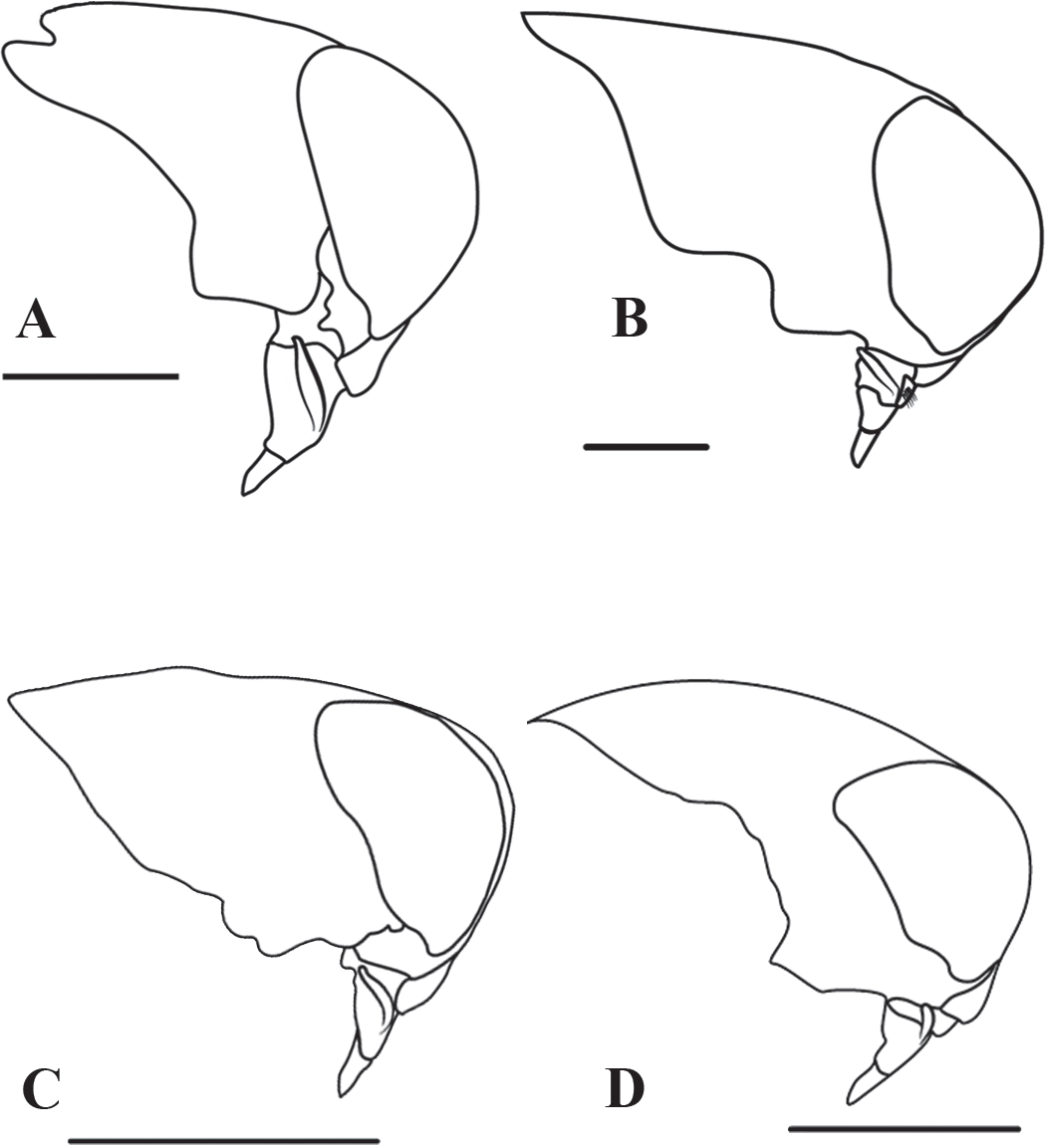
*Buenoa*, male head, lateral view **A***Buenoafemoralis***B***B.amnigenus***C***B.salutis***D***B.fuscipennis*. Scale bars: 1 mm.

### ﻿New records

**Subfamily Anisopinae**

***Buenoa* Kirkaldy, 1904**

#### ﻿ Buenoa amnigenus

Taxon classificationAnimalia

(White, 1879)

1ED0B973-7287-5943-92BA-DC5E7237781A

Fig. 10A

Anisops amnigenus White, 1879: 271 [original description].
Buenoa amnigenus ; Kirkaldy (1904): 120 [genus transfer].

##### Diagnosis.

Small species, male length 4.8–5.4 mm. Synthlipsis very narrow, < 1/10 of vertex width. Labrum sided by pair of tufts of setae. Labial prong slightly longer than third labial article, originating proximally on article. Fore femur without stridulatory area.

##### Taxonomic notes.

A member of the *salutis* group. It differs from the most similar species, *B.amnigenoidea* Nieser, 1970, by the chaetotaxy of the labrum and minor characteristics of the fore trochanter, femur, and tibia. Also, from *B.amnigenopsis* Nieser, 1975 by the presence of a notch on the apex of the fore femur of this species, which is absent in *B.amnigenus*. Finally, it can be distinguished from *B.salutis* based on the larger size, narrower synthlipsis and more pronounced tylus. More detailed comparisons between species of this group can be found in Barbosa and Nessimian (2013a).

##### Distribution.

Argentina (Bachmann 1971). Bolivia (Truxal 1953). Brazil: AL [new record], AM (White 1879; Truxal 1953; Nieser 1975; Barbosa and Nessimian 2013a; Barbosa and Rodrigues 2013), BA [new record], CE (Truxal 1953; Nieser 1975; this work), GO (Truxal 1957), MG (Valbon et al. 2021), MS (Truxal 1953), MT (Heckman 1998), PA (Truxal 1953; Barbosa and Nessimian 2013a), PB (Truxal 1953; Nieser 1975), PE (Truxal 1953; Nieser 1975; this work), PI [new record], RN (Truxal 1953; Nieser 1975), SP (Castanhole et al. 2013; Pereira et al. 2015a), TO (Truxal 1957). Guyana (Truxal 1953). Paraguay (Truxal 1953). Peru (Truxal 1953). Suriname (Nieser 1975). Trinidad & Tobago (Nieser and Alkins-Koo 1991).

##### Material examined.

Brazil, !AL, 2018: 1 ♂: Campo Grande municipality, açude, AL6 station, 07.VII.2018. -9.9436, -36.8275. C.F.B. Floriano, J.M.S. Rodrigues & J.F. Barbosa cols., CEIOC 81609; 1 ♂, 1 ♀: Piaçabuçu municipality, Rio Piauí, 02.V.2018. -10.347, -36.4828. C.F.B. Floriano, J.M.S. Rodrigues & O.M. Magalhães cols., CEIOC 81703. !BA, 2018: 1 ♂: Lençóis municipality, PNCD, Ribeirão do Meio, Terra 9 station, 341 m, 22.VIII.2018. -12.586, -41.3817. C.C. Gonçalves col., CEIOC 81907. CE, 2019: 2 ♂: Aiuaba municipality, EEA, casa da gameleira, Terra 1 station, light trap, 517 m, 09.IV.2019. -6.6976, -40.2812. J.M.S. Rodrigues col., CEIOC 81901; 1 ♂, 1 ♀: same data, except: Sítio Volta de Cima, sede do ICMBIO, Terra 4 station, light trap, 460 m, 10.IV.2019. -6.6025, -40.1286, CZMA; 1 ♂: same data, except: Sítio boa Vista, Terra 6 station, light trap, 583 m, -6.7256, -40.2226, CZMA; 1 ♂: same data, except: Terra 8 station, light trap, 650 m, 11.IV.2019. -6.7425, -40.3065. C.C. Gonçalves col., CZMA; 1 ♂, 1 ♀: same data, except: Sítio do Martins, Terra 10 station, light trap, 476 m, 12.IV.2019. -6.6667, -40.1750. J.S. Prando col., CEIOC 81899; 1 ♂: same data, except: C.C. Gonçalves col., CEIOC 81898; 1 ♂, 1 ♀: same data, except: J.M.S. Rodrigues col., CZMA; 1 ♂, 15 ♀: same data, except: casa da gameleira, Água 1 station, 532 m, 09.IV.2019. -6.6992, -40.2908, CEIOC 81900; 2 ♂, 3 ♀: same data, except: Açude do Letreiro, Água 12 station, 475 m, 13.IV.2019. -6.6163, -40.1545, CEIOC 81902; 2 ♂, 3 ♀: same data, except: CEIOC 81902. Same state, 2021: 6 ♂, 4 ♀: same data, except: estrada, Terra 3 station, light trap, 505 m, 08.VI.2021. -6.6974, -40.2819, CEIOC 81906; 7 ♂, 1 ♀: same data, except: C.C. Gonçalves col., CEIOC 82012; 2 ♂, 1 ♀: same data, except: Terra 5 station, light trap, 493 m, 03.VI.2021. -6.7003, -40.1919, CZMA; 6 ♂, 2 ♀: same data, except: estrada, Terra 11 station, light trap, 634 m, 05.VI.2021. -6.7259, -40.3065. C.C. Gonçalves col., CZMA; 2 ♂, 2 ♀: same data, except: estrada próxima ao riacho, Terra 16 station, light trap, 498 m, 06.VI.2021. -6.6872, -40.2683. CZMA; 1 ♂, 4 ♀: same data, except: Terra 19 station, light trap, 634 m, 07.VI.2021. -6.7259, -40.3065, CZMA; 1 ♂: same data, except: C.C. Gonçalves col., CZMA; 1 ♂, 1 ♀: same data, except: Sítio Jatobá, açude, Água 1 station, 571 m, 06.VI.2021. -6.7351, -40.2424. J.M.S. Rodrigues & F.F.F. Moreira cols., CZMA; 3 ♂, 2 ♀: same data, except: Sítio Volta de Cima, lago, Água 4 station, 434 m, 05.VI.2021. -6.6019, -40.1246, CZMA; 16 ♂, 15 ♀, 4 nymphs: same data, except: Sítio Volta de Baixo, açude, Água 8 station, 431 m, 06.VI.2021. -6.6263, -40.1339, CEIOC 81905; 7 ♂, 7 ♀: same data, except: braço de rio, açude, Água 11 station, 513 m, 07.VI.2021. -6.6997, -40.1917, CZMA. PE, 2019: 2 ♂, 1 ♀: Tupanatinga municipality, PNCA, Fazenda Laranjeiras, Terra 18 station, light trap, 610 m, 20.III.2019. -8.4618, -37.3273. J.M.S. Rodrigues col., CZMA; 2 ♂, 3 ♀: same data, except: CEIOC 81904. !PI, 2020: 1 ♂, 1 ♀: Caracol municipality, PNSC, estrada próxima do Povoado Capim, Terra 15 station, light trap, 17.II.2020. -9.2149, -43.5034. A.A. Alves col., CEIOC 81903.

#### ﻿ Buenoa femoralis

Taxon classificationAnimalia

(Fieber, 1851)

D46207D7-7FED-598D-A242-06D81185B05C

Fig. 10B

Anisops femoralis Fieber, 1851: 483 [original description].
Buenoa femoralis ; Kirkaldy (1904): 120 [genus transfer].

##### Diagnosis.

Large species, male length 7.8–8.6 mm. Synthlipsis wide, nearly 1/2 of vertex width. Labial prong much longer than third labial article, originating slightly after half of article. Fore femur slightly widened at apex, with ~ 17 ridges in a triangular stridulatory area.

##### Taxonomic notes.

*Buenoafemoralis* is one of the largest species of the genus in Brazil, together with *B.antigoneantigone*, *B.crassipes* (Champion, 1901), and *B.oreia* Nieser, Melo, Pelli & Barbosa, 1997. It is usually larger than the first two species and smaller than the last one, and shows different shapes of the fore femur, stridulatory area, and labial prong. The closest species in northeastern Brazil is *B.pallipes*, which is a distinctly smaller species, with differently shaped labial prong.

##### Distribution.

Brazil: AM (Barbosa and Nessimian 2013a), PI [new record], PR (Jaczewski 1928). Peru (Truxal 1953). Puerto Rico (Fieber 1851). U.S. Virgin Islands (Barber 1939).

##### Material examined.

Brazil, !PI, 2018: 1 ♂, 2 ♀, 4 nymphs: Caracol municipality, PNSC, entre o Mirante “Janela do Sertão” e o cemitério, Água 2 station, 566 m, 10.XII.2018. -9.2188, -43.4902. J.M.S. Rodrigues & O.M. Magalhães cols., CEIOC 82007; 1 ♂: same data, except: Guaribas municipality, açude, Água 10 station, 640 m, 14.XII.2018. -9.3649, -43.7546, CEIOC 82006; 1 ♂, 2 ♀, 1 nymph: same data, except: Caracol municipality, Água 13 station, 526 m, 15.XII.2018. -9.2140, -43.4965, CZMA; 2 ♂: same data, except: proximo a estrada, Terra 6 station, 11.XII.2018. -9.2137, -43.4986. C.C. Gonçalves col., CEIOC 82080. Same state, 2020: 4 ♂, 2 ♀: same data, except: poças ao lado da estrada proximo ao cemitério, Água 4 station, 565 m, 13.II.2020. -9.2185, -43.4904. J.M.S. Rodrigues & I.R.S. Cordeiro cols., CEIOC 81910; 1 ♂: same data, except: Povoado Sobrado, alagado em área de pasto, Água 11 station, 499 m, 16.II.2020. -9.2111, -43.5202, CEIOC 81908; 1 ♂, 8 ♀: same data, except: Guaribas municipality, brejão, açude, Água 12 station, 640 m, 18.II.2020. -9.3650, -43.7544, CEIOC 81909.

#### ﻿ Buenoa fuscipennis

Taxon classificationAnimalia

(Berg, 1879)

AA31CBDC-2645-5A84-8B94-039AF95D41CC

Fig. 10C

Anisops fuscipennis Berg, 1879: 198–199 [original description].
Anisops naias Kirkaldy, 1899e: 194 [synonym; Truxal (1953): 1460].
Buenoa fuscipennis ; Kirkaldy (1904): 120 [genus transfer].
Buenoa dentipes Jaczewski, 1928: 127 [synonym; Truxal (1953): 1460].

##### Diagnosis.

Medium to large species, male length 6.8–7.2 mm. Synthlipsis wide, slightly < 1/2 of vertex width. Labial prong shorter than third labial article, originating proximally on article. Fore trochanter with a conspicuous process pointing backwards. Fore femur without stridulatory area. Middle tibia widened at apex.

##### Distribution.

Argentina (Berg 1879). Bolivia (Truxal 1953). Brazil: CE [new record], MT (Heckman 1998), PA (Barbosa and Nessimian 2013a; Nobre et al. 2019), PE [new record], PR (Jaczewski 1928), SC (Truxal 1953). Chile (Kirkaldy 1899a). Paraguay (Truxal 1953). The record from Venezuela (Kirkaldy 1904) is questionable.

##### Material examined.

Brazil, !CE, 2019: 4 ♂, 2 ♀: Aiuaba municipality, EEA, Sítio Jatobá, açude alagado, Água 8 station, 572 m, 11.IV.2019. -6.7346, -40.2429. J.M.S. Rodrigues col., CEIOC 81922; 2 ♂, 1 ♀: same data, except: Sítio Volta de Baixo, Terra 12 station, light trap, 441 m, 14.IV.2019. -6.6254, -40.1336, CZMA. !PE, 2019: 4 ♂: Tupanatinga municipality, PNCA, Fazenda Brejo de São José, poça temporária, Água 8 station, 700 m, 16.III.2019. -8.506, -37.2237. H. Rodrigues & J.M.S. Rodrigues cols., CEIOC 82004; 1 male, 3 ♀: same data, except: Fazenda Laranjeiras, poça temporária ao lado da estrada, Água 9 station, 325 m, 17.III.2019. -8.5002, -37.3111, CZMA; 10 ♂: same data, except: Chapadão, poça temporária, Água 18 station, 957 m, 20.III.2019. -8.5244, -37.2394, CEIOC 82003; 1 ♂, 4 ♀: same data, except: Buíque municipality, trilha para Sítio Arqueológico Alcobaça, Terra 3 station, light trap, 658 m, 15.III.2019. -8.5274, -37.1945, CZMA; 1 ♂, same data, except: Fazenda do Brejo, Terra 7 station, light trap, 706 m, 16.III.2019. -8.5199, -37.2249, CZMA; 3 ♂, 1 ♀: same data, except: Tupanatinga municipality, estrada do gado, Terra 14 station, light trap, 19.III.2019. -8.4866, -37.3404, CEIOC 82005. Same state, 2021: 2 ♂: same data, except: Barro branco, Comunidade Muquém, açudes, Água 1 station, 824 m, 10.III.2021. -8.5001, -37.3113. J.M.S. Rodrigues & R. Jordão cols., CEIOC 81923.

#### ﻿ Buenoa koina

Taxon classificationAnimalia

Nieser & Pelli, 1994

6DFEEC39-566B-5B0C-8A68-D6398ACFE5F0

Fig. 10D

**Figure 8.**
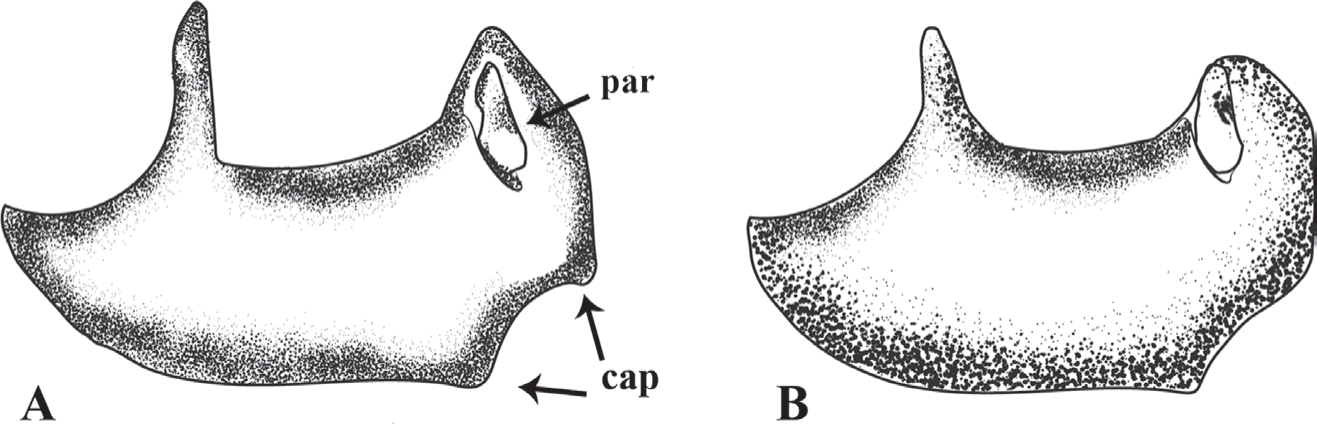
*Notonecta*, male genital capsule, lateral view, modified from Hungerford 1933**A***Notonectadisturbata***B***N.pulchra*. Abbreviations: cap = caudoventral protuberance, par = paramere.

**Figure 9.**
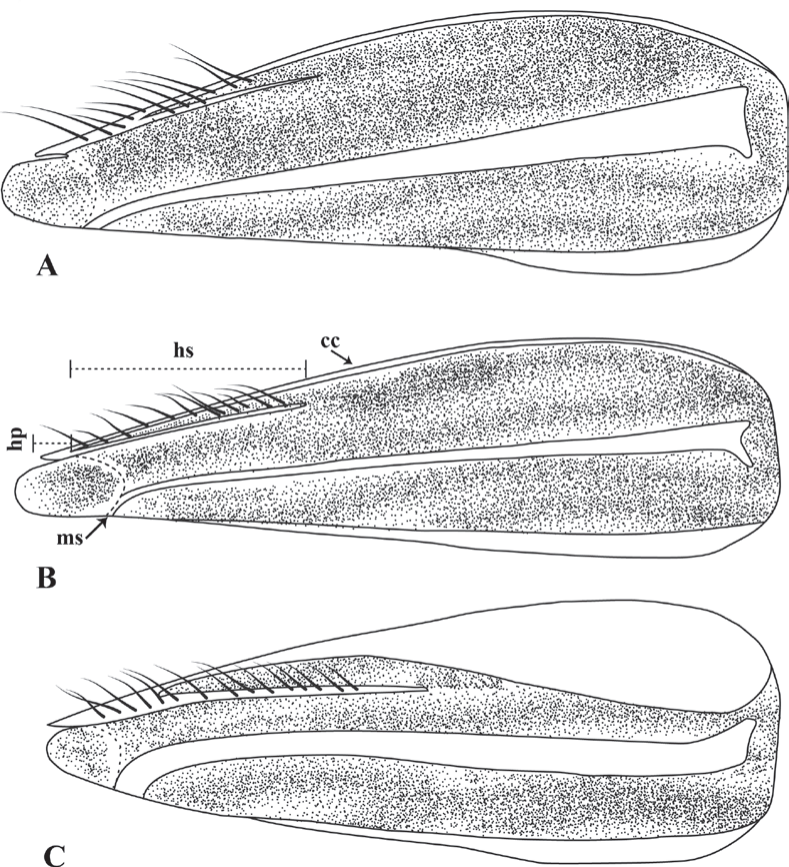
*Martarega*, aspect of brachypterous male hemelytra, lateral view; hyaline portions are represented as undotted, modified from Barbosa 2014**A***Martaregabentoi***B***M.brasiliensis***C***M.membranacea*. Abbreviations: cc = claval commissure, hp = hemelytral process, hs = hemelytral process stripe, ms = medial longitudinal stripe.

**Figure 10.**
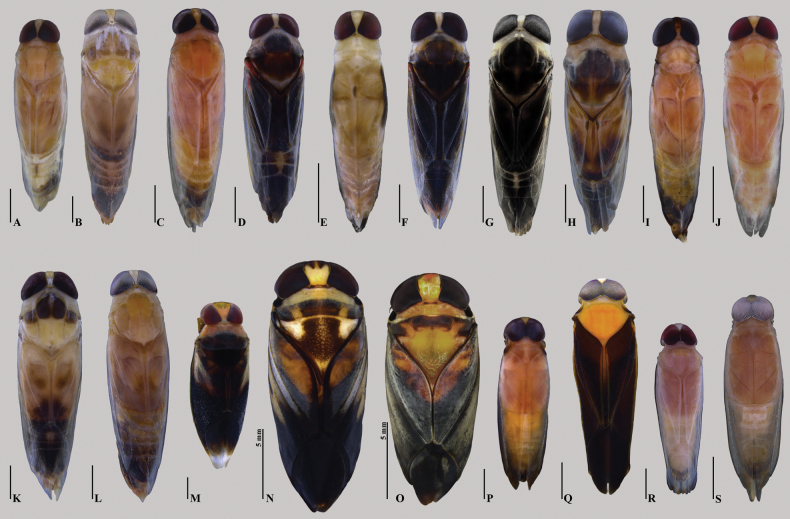
Notonectidae, dorsal habitus of species found in this survey, male specimens **A***Buenoaamnigenus***B***B.femoralis***C***B.fuscipennis***D***B.koina***E***B.konta***F***B.mutabilis***G***B.pallipes***H***B.platycnemis***I***B.pseudomutabilis***J***B.salutis***K***B.tarsalis***L***B.unguis***M***Notonectadisturbata***N***Enitharoidesbrasiliensis***O***E.tricomerus***P, Q***Martaregabentoi***R***M.brasiliensis***S***M.membranacea*. **A–D, F–H, J–O, Q–R** are macropterous specimens; **E, I, P, S** are brachypterous specimens. Scale bars: 1 mm, unless otherwise indicated.

Buenoa koina Nieser & Pelli, 1994: 3, 4, figs 8–10 [original description].

##### Diagnosis.

Medium-sized species, male length ~ 5.9 mm. Synthlipsis wide, slightly > 1/2 of vertex width. Labial prong subequal to slightly shorter than third labial article, originating proximally on article. Fore femur with weak stridulatory area containing 4–6 ridges.

##### Taxonomic notes.

*Buenoakoina* is one of smallest species of the genus with wide synthlipsis. It can be distinguished from the most similar species, *B.pallens* (Champion, 1901) and *B.pallipes* (Fabricius, 1803), by the pronotum not tricarinate and the shorter length of the labial prongthan in the two aforementioned species.

##### Distribution.

Brazil: BA [new record], MG (Nieser and Pelli 1994; Nieser and Melo 1997; Carrenho et al. 2020).

##### Material examined.

Brazil, !BA, 2018: 4 ♂, 1 ♀: Mucugê municipality, PNCD, Água 3 station, 19.VIII.2018. -12.9908, -41.3507. J.M.S. Rodrigues col., CEIOC 81921. Same state, 2021: 1 ♂, 2 ♀: same data, except: Palmeiras municipality, Rio Preto, Água 1 station, 820 m, 05.V.2021. -12.6036, -41.5250. J.M.S. Rodrigues & J.F. Barbosa cols., CEIOC 81920.

#### ﻿ Buenoa konta

Taxon classificationAnimalia

Nieser & Pelli, 1994

52F110C9-E487-5FE1-8CBB-680E2FC96208

Fig. 10E

Buenoa konta Nieser & Pelli, 1994: 1–3, figs 1–4 [original description].

##### Diagnosis.

Very small species, male length 3.7–4.3 mm. Synthlipsis narrow, 1/4 of vertex width). Labial prong subequal in length to third labial article, originating proximally on article. Blackish brown hoof print-like pattern at hemelytra in dorsal view. Fore femur with wide apex, without stridulatory area.

##### Taxonomic notes.

*Buenoakonta* and *B.salutis* are the two smallest species of *Buenoa* occurring in Brazil. The former has a conspicuous blackish brown hoof print-like pattern on the hemelytra in dorsal view. This mark, with other morphological features like the short labial prong and the wide apex of the fore femur, ensures an easy identification.

##### Distribution.

Brazil: AL [new record], BA [new record], CE [new record], GO (Barbosa and Dias-Silva 2017), MG (Nieser and Pelli 1994; Nieser and Melo 1997; Melo and Nieser 2004; Barbosa and Rodrigues 2013; Carrenho et al. 2020), MS (Barbosa et al. 2010c), PA (Barbosa et al. 2010a; Barbosa and Nessimian 2013a), RJ (Ribeiro et al. 2010), SE [new record].

##### Material examined.

Brazil, !AL, 2018: 1 ♂: Maragogi municipality, APACC, APACC2 station, 29.IV.2018. -8.922, -35.1796. C.F.B. Floriano, J.M.S. Rodrigues & O.M. Magalhães cols., CEIOC 81700; 1 ♂, 2 nymphs: same data, except: Murici municipality, EEM, EEM5 station, 28.IV.2018. -9.2540, -35.8010, CEIOC 81698. !BA, 2021: 2 ♂, 6 ♀, 1 nymph: Andaraí municipality, PNCD, Rio Garapa, Água 5 station, 08.V.2021. -12.7456, -41.3453. J.M.S. Rodrigues & J.F. Barbosa cols., CEIOC 81915. !CE, 2021: 2 ♂: Aiuaba municipality, EEA, sítio Volta de Cima, lago, Água 4 station, 434 m, 05.VI.2021. -6.6019, -40.1246. J.M.S. Rodrigues col., CEIOC 81916. !SE, 2018: 4 ♂, 8 ♀, 5 nymphs: Areia Branca municipality, PNSI, Riacho Negro, PNSI2 station, 08.VII.2018. -10.7475, -37.3402. C.F.B. Floriano, J.F. Barbosa & J.M.S. Rodrigues cols., CEIOC 81621.

#### ﻿ Buenoa mutabilis

Taxon classificationAnimalia

Truxal, 1953

15DAE9C7-F3C3-58AB-AC75-FB90DF77F1C8

Fig. 10F

Buenoa mutabilis Truxal, 1953: 1432–1435, fig. 61 [original description].

##### Diagnosis.

Medium-sized species, male length 5.2–6.0 mm. Synthlipsis narrow, ~ 1/3 of vertex width. Labial prong with variable shape, longer than third labial article, originating proximally on article. Fore femur not widened at apex; stridulatory area subtriangular, with 10–18 ridges.

##### Distribution.

Brazil: CE [new record], GO (Truxal 1957), MG (Truxal 1957; Nieser and Melo 1997; Vianna and Melo 2003; Melo and Nieser 2004), PI (Takiya et al. 2016; this work), SE [new record]. Guyana (Truxal 1953). Haiti (Truxal 1953). Paraguay (Truxal 1953). Peru (Truxal 1953). U.S. Virgin Islands (Rogers and Cruz-Rivera 2021). Venezuela (Truxal 1953).

##### Material examined.

Brazil, !CE, 2021: 3 ♂, 2 ♀: Aiuaba municipality, EEA, estrada, Terra 3 station, light trap, 505 m, 08.VI.2021. -6.6974, -40.2819. J.M.S. Rodrigues col., CEIOC 82001; 1 ♂, 2 ♀: same data, except: estrada próximo ao riacho, Terra 16 station, light trap, 498 m, 06.VI.2021. -6.6871, -40.2683, CZMA; 18 ♂, 11 ♀: same data, except: açude/riacho, Água 3 station, 507 m, 04.VI.2021. -6.6921,-40.1871. J.M.S. Rodrigues & C.C. Gonçalves cols., CEIOC 81914. PI, 2018: 9 ♂, 12 ♀: Guaribas municipality, PNSC, poça próxima ao “Museu do Vaqueiro”, 525 m, 11.XII.2018. -9.1379, -43.5972. J.M.S. Rodrigues & O.M. Magalhães cols., CEIOC 82002. !SE, 2018: 2 ♂, 1 ♀, 4 nymphs: Areia Branca municipality, Riacho Vermelho, PNSI1 station, 08.VII.2018. -10.7387, -37.33547. C.F.B. Floriano, J.M.S. Rodrigues & J.F. Barbosa cols., CEIOC 81681.

#### ﻿ Buenoa pallipes

Taxon classificationAnimalia

(Fabricius, 1803)

4FBBEF2C-83E8-59E8-8F1D-064C124F6AB4

Fig. 10G

Notonecta pallipes Fabricius, 1803: 103 [original description].
Anisops pallipes ; Stål (1868): 137 [genus transfer].
Buenoa pallipes ; Kirkaldy (1904): 123 [genus transfer].

##### Diagnosis.

Medium-sized species, male length 5.5–6.2 mm. Synthlipsis wide, slightly < 1/2 of vertex width. Labial prong arc-shaped, distinctly longer than third labial article, originating distally on article. Fore femur apex widened; stridulatory area subtriangular, with 17 ridges.

##### Taxonomic notes.

Another medium-sized species, *B.pallipes* is similar to *B.mutabilis* and *B.platycnemis* (Fieber 1851). However, it has the apex of the fore femur wider and the labial prong longer than in the former species, and the labial prong more parallel to the axis of the frons than in the latter.

##### Distribution.

Barbados (Nieser 1967). Bolivia (Kirkaldy 1899d). Brazil: AL [new record] AM (Nieser 1970), PA (Nieser 1970), PE [new record]. Colombia (Kirkaldy 1899c). Costa Rica (de la Torre-Bueno 1906). Cuba (Nieser 1967). Ecuador (Kirkaldy 1899c). Guadeloupe (Kirkaldy 1904). Hawaiian Islands (Kirkaldy 1910). Honduras (Truxal 1953). Jamaica (Kirkaldy 1900). Martinique (Kirkaldy 1904). Mexico (Champion 1901). Panama (Kirkaldy 1899b). Paraguay (Truxal 1953). Peru (Truxal 1953). Puerto Rico (Fieber 1851). St. Barthélemy (Lethierry 1881). St. Vincent & The Grenadines (Uhler 1893). U.S. Virgin Islands (Fieber 1851). Records from North America north of Mexico are erroneous.

##### Material examined.

Brazil, !AL, 2018: 1 ♂, 1 ♀: Quebrângulo municipality, RBPT, Sítio Juliana, brejo, PPT4 station, 04.VII.2018. -9.2608, -36.4157. C.F.B. Floriano, J.M.S. Rodrigues and J.F. Barbosa cols., CEIOC 81705. !PE, 2019: 5 ♂, 11 ♀, 6 nymphs: Tupanatinga municipality, PNCA, poço, aproximadamente 400 m da casa dos brigadistas, Água 1 station, 818 m, 14.III.2019. -8.5725, -37.2367. H. Rodrigues & J.M.S. Rodrigues col., CEIOC 82011. Same state, 2021: 2 ♂, 2 ♀: same data, except: Buíque municipality, olho d’água do pico, Água 10 station, 763 m, 15.III.2021. -8.5577, -37.1951. J.M.S. Rodrigues & R. Jordão cols., CEIOC 82079; 20 ♂, 16 ♀: same data, except: Tupanatinga municipality, nascente do ICMBIO, brejo, Água 11 station, 807 m, 16.III.2021. -8.5724, -37.2368, CEIOC 81918.

#### ﻿ Buenoa platycnemis

Taxon classificationAnimalia

(Fieber, 1851)

6AD6274E-1B5C-5B65-88FA-E11B38506F8F

Fig. 10H

Anisops platycnemis Fieber, 1851: 485 [original description].
Buenoa platycnemis ; Kirkaldy (1904): 123 [genus transfer].

##### Diagnosis.

Medium to large species, male length 4.6–5.4 mm. Synthlipsis narrow, slightly < 1/2 of vertex width. Labial prong straight, much longer than third labial article, originating distally on article. Fore femur with apex widened; stridulatory area subtriangular, with 11–14 ridges.

##### Taxonomic notes.

Similar to other medium-sized species, like the aforementioned *B.mutabilis* and *B.pallipes*. A thorough examination of the morphological characteristics described for each of these species is necessary for their correct identification.

##### Distribution.

Argentina (Kirkaldy 1904). Bonaire (Nieser 1967). Brazil: AM (Barbosa and Nessimian 2013a), GO (Truxal 1957), MA (Truxal 1953), MT (Nieser 1970), PA (Nieser 1970; Barbosa and Nessimian 2013a; Nobre et al. 2019), PE [new record], TO (Truxal 1957), RJ (Kirkaldy 1904; Ribeiro et al. 1998, 2010; Nessimian and Ribeiro 2000; Barbosa et al. 2010c), SE [new record]. Cayman Islands (Truxal 1953). Colombia (Truxal 1953). Costa Rica (Truxal 1953). Cuba (Uhler 1884). Curacao (Nieser 1967). Guadeloupe (Nieser 1967). Haiti (Truxal 1953). Jamaica (Truxal 1953). Martinique (Truxal 1953). Mexico (Uhler 1884). Nicaragua (López et al. 1998). Panama (Truxal 1953). Peru (Truxal 1953). Puerto Rico (Fieber 1851). St. Martin (Nieser 1967). Trinidad & Tobago (Nieser and Alkins-Koo 1991). United States (Uhler 1884). Venezuela (Truxal 1953). U.S. Virgin Islands (Fieber 1851).

##### Material examined.

!PE, 2019: 1 ♂: Tupanatinga municipality, PNCA, Riacho de Leís, poças temporárias ao longo do leito, Água 14 station, 750 m, 18.III.2019. -8.5829, -37.2445. J.M.S. Rodrigues & H. Rodrigues cols., CEIOC 81919. !SE, 2018: 1 ♂: Estância municipality, Complexo de Dunas da Praia do Saco, REBIOSI17 station, 05.V.2018. -11.4190, -37.3223. C.F.B. Floriano, J.M.S. Rodrigues & O.M. Magalhães cols., CEIOC 81624; 1 ♂: same data, except: Canhoba municipality, açude, SE1 station, 07.VII.2018. -10.1287, -36.9698. C.F.B. Floriano, J.M.S. Rodrigues & J.F. Barbosa cols., CEIOC 81627.

#### ﻿ Buenoa pseudomutabilis

Taxon classificationAnimalia

Barbosa, Ribeiro & Nessimian, 2010

C321E7BA-DB5A-5427-807E-93E86F7C4292

Fig. 10I

Buenoa pseudomutabilis Barbosa, Ribeiro & Nessimian, 2010: 561–564, figs 1, 10–14 [original description].

##### Diagnosis.

Medium-sized species, male length 5.8–6.0 mm. Synthlipsis narrow, ~ 2/5 of vertex width. Tylus inflated and pubescent. Labial prong longer than third labial article, originating proximally on article. Fore femur narrowed at apex; stridulatory area subtriangular, with 14 ridges. Dark nodule on abdominal ventral laterotergite I.

##### Taxonomic notes.

*Buenoapseudomutabilis* greatly resembles *B.mutabilis*, hence the specific name. This species differs from *B.mutabilis* in the lower width of head/anterior width of vertex ratio (5.0–5.4 in *B.pseudomutabilis*, 6.5 in *B.mutabilis*) and the fewer teeth on the stridulatory comb of the fore tibia (21–25 teeth in *B.pseudomutabilis*, 33–38 in *B.mutabilis*).

##### Distribution.

Brazil: BA [new record], GO (Barbosa and Dias-Silva 2017), PI (Takiya et al. 2016), RJ (Barbosa et al. 2010c).

##### Material examined.

Brazil, !BA, 2018: 1 ♂: Mucugê municipality, PNCD, Água 11 station, 23.VIII.2018. -13.2932, -41.2414. J.M.S. Rodrigues & F.F.F Moreira cols., CEIOC 81917.

#### ﻿ Buenoa salutis

Taxon classificationAnimalia

Kirkaldy, 1904

8CFD2033-8B4A-5A0D-AE0E-DE0E3C23DD63

Fig. 10J

Buenoa salutis Kirkaldy, 1904: 124 [original description].
Buenoa mallochi Jaczewski, 1928: 129–130, figs 23–27 [synonym; Truxal (1953): 1469].

##### Diagnosis.

Small species, male length 3.4–4.0 mm. Synthlipsis narrow, ~ 1/10 tenth of vertex width. Labial prong subequal to slightly longer than third labial article, originating medially on article. Fore femur narrowed at apex, without stridulatory area.

##### Distribution.

Argentina (Bachmann 1962). Bolivia (Truxal 1953). Brazil: AL [new record], AM (Truxal 1953; Nieser 1970; Barbosa and Nessimian 2013a), BA [new record], CE (Truxal 1953; this work), MG (Nieser and Melo 1997; Melo and Nieser 2004; Barbosa and Rodrigues 2013), MS (Floriano et al. 2013), MT (Heckman 1998), PA (Truxal 1953; Nieser 1970; Barbosa and Nessimian 2013a), PB (Truxal 1953), PE (Truxal 1953), PI (Takiya et al. 2016), PR (Jaczewski 1928), RJ (Ribeiro et al. 1998, 2010), RR (Barbosa and Nessimian 2013a), RS (Truxal 1953; Kleerekoper 1955), SE [new record], SP (Truxal 1953), TO (Truxal 1957). Colombia (Roback and Nieser 1974). French Guiana (Kirkaldy 1904). Guyana (Truxal 1953). Paraguay (Truxal 1953). Suriname (Nieser 1968). Trinidad & Tobago (Nieser and Alkins-Koo 1991). Venezuela (Truxal 1953).

##### Material examined.

Brazil, !AL, 2018: 3 ♂, 2 ♀, 3 nymphs: Maragogi municipality, APACC, APACC2 station, 29.IV.2018. -8.922, -35.1795. C.F.B. Floriano, J.M.S. Rodrigues & O.M. Magalhães cols., CEIOC 81695; 1 ♂, 2 ♀: same data, except: Piaçabuçu municipality, APAP, Rio Piauí, 02.V.2018. -10.3470, -36.4828, CEIOC 81697; 1 ♂: same data, except: Feliz Deserto municipality, APAP, APAP3 station, -10.3252, -36.3506, CEIOC 81696. Same state, 2019: 1 ♂, 1 ♀: Marimbondo municipality, Flexeiras, açude, ALSE11 station, 122 m, 23.V.2019. -9.5074, -36.2340. J.M.S. Rodrigues, W. Souza & F.F.F. Moreira cols., CEIOC 81955. !BA, 2018: 2 ♂, 1 ♀: Lençóis municipality, PNCD, Ribeirão do Meio, Terra 9 station, 341 m, 22.VIII.2018. -12.586, -41.3817. J.S. Prando col., CEIOC 81912. CE, 2019: 2 ♂, 1 ♀: Aiuaba municipality, EEA, casa da gameleira, Terra 1 station, light trap, 517 m, 09.IV.2019. -6.6976, -40.2812. J.M.S. Rodrigues col., CEIOC 81913; 1 ♂: same data, except: Sítio boa Vista, Terra 6 station, light trap, 583 m, 10.IV.2019. -6.7256, -40.2226, CZMA; 1 ♂: same data, except: Sítio do Martins, Terra 10 station, light trap, 476 m, 12.IV.2019. -6.6667, -40.1750. J.S. Prando col., CZMA. Same state, 2021: 15 ♂, 12 ♀: same data, except: estrada, Terra 11 station, light trap, 634 m, 05.VI.2021. -6.7259, -40.3065. J.M.S. Rodrigues col., CEIOC 81911. !SE, 2018: 1 ♂, 2 ♀: Areia Branca Municipality, PNSI, Riacho Negro, PNSI2 station, 08.VII.2018. -10.7475, -37.34025. C.F.B. Floriano, J.F. Barbosa & J.M.S Rodrigues cols., CEIOC 81610.

#### ﻿ Buenoa tarsalis

Taxon classificationAnimalia

Truxal, 1953

07FA7AB6-C5AC-5CAB-9020-1E5F0EDFE7C5

Fig. 10K

Buenoa tarsalis Truxal, 1953: 1392–1395, fig. 49 [original description].

##### Diagnosis.

Medium to large species, male length 6.2–7.2 mm. Synthlipsis wide, ~ 1/2 of vertex width. Labial prong longer than third labial article, originating proximally on article. Fore femur with apex narrowed; stridulatory area oblong, with 17–23 ridges. First article of middle tarsus deeply emarginated.

##### Taxonomic notes.

*Buenoatarsalis* resembles *B.femoralis* but has a very conspicuous emargination on the first article of the middle tarsus, which ensures its correct identification.

##### Note.

The most abundant and widespread species in this survey, present in all studied states.

##### Distribution.

Brazil: AL [new record], AM (Nieser 1970; Barbosa and Nessimian 2013a), BA [new record], CE (Truxal 1953; this work), GO (Barbosa and Dias-Silva 2017), MG (Nieser and Melo 1997; Melo and Nieser 2004; Gutiérrez et al. 2017a, b), PA (Truxal 1953; Barbosa and Nessimian 2013a), PB (Truxal 1953), PE (Truxal 1953; this work), PI (Takiya et al. 2016; this work), RJ (Truxal 1953; Barbosa et al. 2010c), RN (Truxal 1953), SE [new record].

##### Material examined.

Brazil, !AL, 2018: 2 ♂, 1 ♀: Campo Grande municipality, açude, AL6 station, 07.VII.2018. -9.9436, -36.8275. C.F.B. Floriano, J.M.S. Rodrigues & J.F. Barbosa cols., CEIOC 81613; 15 ♂, 14 ♀: same data, except: rio, AL7 station, -9.9559, -36.8373, CEIOC 81611; 3 ♂, 3 ♀, 1 nymph, same data, except: Coité do Noia municipality, Estrada AL-110, Rio Coruripe, AL5 station, 06.VII.2018. -9.6987, -36.5845, CEIOC 81684; 1 ♂: same data, except: Maragogi municipality, APACC, APACC2 station, 29.IV.2018. -8.922, -35.1795, C.F.B. Floriano, J.M.S. Rodrigues & O.M. Magalhães cols., CEIOC 81614; 9 ♂, 9 ♀ 19 nymphs: same data, except: Quebrângulo municipality, RBPT, sítio Juliana, brejo, PPT4 station, 04.VII.2018. -9.2608, -36.4157. C.F.B. Floriano, J.M.S. Rodrigues & J.F. Barbosa cols., CEIOC 81683. Same state, 2019: 13 ♂, 12 ♀, 9 nymphs: Marimbondo flexeiras municipality, açude, ALSE11 station, 122 m, -9.5074, -36.2340. J.M.S. Rodrigues, W. Souza & F.F.F. Moreira cols., CEIOC 82066; 16 ♂, 14 ♀, 3 nymphs: same data, except: Minador do Negrão municipality, açude, ALSE13 station, 253 m, 23.V.2019. -9.3679, -36.8425, CEIOC 82073. !BA, 2018: 1 ♂: Lençóis municipality, PNCD, Ribeirão do Meio, Terra 9 station, 341 m, 22.VIII.2018. -12.586, -41.3817. J.S. Prando col., CEIOC 82039. CE, 2019: 1 ♂, 9 nymphs: Aiuaba municipality, EEA, Sítio do Martins, casa da gameleira, Água 1, 532 m, 09.IV.2019. -6.6992, -40.2908. J.M.S. Rodrigues col., CZMA; 2 ♂, 3 ♀: same data, except: área alagada ao lado da estrada para a casa do cajueiro, Água 4 station, 508 m, 10.IV.2019. -6.6923, -40.1873, CEIOC 81996; 2 ♂, 5 ♀, 7 nymphs: same data, except: Sítio Boa Vista, área alagada, Água 7 station, 570 m, 11.IV.2019. -6.7255, -40.2226, CEIOC 82075; 2 ♂, 8 ♀: same data, except: Sítio Jatobá, açude alagado, Água 8 station, 572 m, -6.7346, -40.2429, CZMA; 3 ♂, 8 ♀, 2 nymphs: same data, except: Vaginha da Gameleira, açude, Água 9 station, 510 m, 12.IV.2019. -6.6866, -40.2625, CZMA; 10 ♂, 6 ♀, 2 nymphs: same data, except: Açude do Letreiro, Água 12 station, 475 m, 13.IV.2019. -6.6163, -40.1545, CEIOC 82000; 3 ♂, 14 ♀, 5 nymphs: Sítio Volta de Baixo, açude cheio, Água 13 station, 431 m, -6.6263, -40.1339, CZMA; 1 ♂: same data, except: Serra da Lagoa, Terra 8 station, light trap, 650 m, 11.IV.2019. V. Quintas col., CEIOC 82072; 3 ♂, 3 ♀: Sítio Volta de Baixo, Terra 12 station, light trap, 441 m, 14.IV.2019. -6.6254, -40.1336. J.M.S. Rodrigues col., CEIOC 81985. Same state, 2021: 1 ♂, 18 ♀, 1 nymph: same data, except: Sítio Jatobá, açude, Água 1 station, 571 m, 03.VI.2021. -6.7351, -40.2424. J.M.S. Rodrigues & F.F.F Moreira cols., CZMA; 10 ♂, 2 ♀: same data, except: brejo, Água 6 station, 570 m, 05.VI.2021. -6.7263, -40.2229, CEIOC 81997; 4 ♂, 31 ♀: same data, except: poça, Água 10 station, 507 m, -6.6866, -40.2625, CZMA; 8 ♂, 4 ♀, 10 nymphs: same data, except: braço de rio, açude, Água 11 station, 513 m, 07.VI.2021. -6.6997, -40.1917, CEIOC 81998; 4 ♂, 6 ♀: same data, except: brejo, Água 12 station, 584 m, -6.7384, -40.2475, CZMA; 7 ♂, 2 ♀: same data, except: estrada, Terra 3 station, light trap, 505 m, 08.VI.2021. -6.6974, -40.2819. J.M.S. Rodrigues col., CZMA; 1 ♂: same data, except: C.C. Gonçalves col., CEIOC 82069; 4 ♂, 2 ♀; same data, except: estrada, Terra 5 station, light trap, 493 m, 03.VI.2021. -6.7003, -40.1919. J.M.S. Rodrigues col., CZMA; 2 ♂, 2 ♀: same data, except: estrada ao lado do brejo, Terra 8 station, light trap, 486 m, 04.VI.2021. -6.6920, -40.1870, CZMA; 2 ♂, 2 ♀: same data, except: estrada, Terra 11 station, light trap, 634 m, 05.VI.2021. -6.7259, -40.3065, CZMA; 5 ♂, 4 ♀: same data, except: estrada prox. ao riacho, Terra 16 station, light trap, 498 m, 06.VI.2021. -6.6871, -40.2683, CEIOC 81999. PE, 2019: 3 ♂, 8 ♀: Tupanatinga municipality, PNCA, caldeirões, aproximadamente 600 m da torre dos brigadistas, poças temporárias, Água 3 station, 783 m, 14.III.2019: -8.5642, -37.2389. H. Rodrigues & J.M.S. Rodrigues cols., CEIOC 82064; 11 ♂, 10 ♀: same data, except: poça temporária na trilha da casa da farinha, Água 4 station, 814 m, -8.5615, -37.2340, CEIOC 82070; 3 ♂, 6 ♀: same data, except: Barreiro do Pititi, poça temporária, Água 5 station, 832 m, -8.5599, -37.2375, CEIOC 82030; 4 ♂: same data, except: Buíque municipality, riacho salgado do início da trilha para o sítio arqueológico Alcobaça, poças temporárias, Água 6 station, 670 m, 15.III.2019. -8.5261, -37.1938. H. Rodrigues & J.M.S. Rodrigues cols., CEIOC 82041; 4 ♂, 20 ♀: same data, except: brejo, perto da sede, poça temporária, Fazenda Brejo de São José, Água 7 station, 699 m, 16.III.2019. -8.5372, -37.2200, CEIOC 82035; 8 ♂: same data, except: poça temporária, Água 8 station, 700 m, -8.506, -37.2237, CEIOC 82034; 34 ♂, 22 ♀: same data, except: Fazenda Laranjeiras, poça temporária ao lado da estrada, Água 9 station, 825 m, 17.III.2019. -8.5002, -37.3111, CEIOC 82031; 1 ♂, 2 ♀, 3 nymphs: same data, except: Fazenda Juá, perto da sede, poça temporária coberta de macrófitas, Água 11 station, 541 m, -8.4116, -37.3601, CEIOC 82029; 2 ♂, 1 ♀, 1 nymph: same data, except: riacho temporário, Água 12 station, 548 m, -8.4350, -37.3467, CEIOC 82027; 4 ♂, 8 ♀: same data, except: Riacho Catimbau, poça temporária ao lado do leito, Água 13 station, 745 m, 18.III.2019. -8.5834, -37.2469, CZMA; 3 ♂, 3 ♀: same data, except: Riacho de Leís, poças temporárias ao longo do leito, Água 14 station, 750 m, -8.5829, -37.2445, CEIOC 82023; 1 ♂, 4 ♀, 8 nymphs: same data, except: Caldeirões da Igrejinha, poças temporárias, Água 17 station, 949 m, 20.III.2019. -8.5024, -37.2543, CEIOC 82022; 1 ♂: same data, except: Chapadão, poça temporária, Água 18 station, 957 m, -8.5244, -37.2394, CZMA; 3 ♂, 1 ♀: same data, except: terreno do ICMBio, Terra 2 station, light trap, 730 m, 17.III.2019. -8.5274, -37.1945. J.M.S. Rodrigues col., CEIOC 82025; 1 ♂, 2 ♀: same data, except: CZMA; 1 ♂: same data, except: trilha para Sítio Arqueológico do Alcobaça, Terra 3 station, light trap, 658 m, 15.III.2019. -8.5274, -37.1945. H. Rodrigues col., CEIOC 82024; 4 ♂, 9 ♀; same data, except: J.M.S. Rodrigues col., CEIOC 82036; 4 ♂: same data, except: Fazenda do brejo, Terra 7 station, light trap, 706 m, 16.III.2019. -8.5199, -37.2249. C.C. Gonçalves & D.M. Takiya cols., CZMA; 12 ♂, 13 ♀: same data, except: J.M.S. Rodrigues col., CEIOC 82042; 5 ♂, 7 ♀: same data, except: estrada do gado, Terra 14 station, light trap, 663 m, 19.III.2019. -8.4866, -37.3404. J.MS. Rodrigues col., CEIOC 82043; 1 ♂, 1 ♀: same data, except C.C. Gonçalves col., CZMA. Same state, 2021: 4 ♂, 6 ♀: Tupanatinga municipality, PNCA, Barro branco, Comunidade Muquém, açudes, Água 1 station, 824 m, 10.III.2021. -8.5001, -37.3113. J.M.S. Rodrigues & R. Jordão cols., CEIOC 81993; 2 ♂, 5 ♀: same data, except: poça ao lado da estrada, Água 2 station, 904 m, -8.4982, -37.2852, CEIOC 81982; 1 ♂, 4 ♀: same data, except: região dos Breús, trilha das umburanas, caldeirão, Água 4 station, 940 m, 13.III.2021. -8.4891, -37.2637, CEIOC 81983; 3 ♂, 1 ♀, 3 nymphs: same data, except: caldeirões em rocha, ao lado de estrada de chão perto da igrejinha, Água 6 station, -8.5024, -37.2543, CEIOC 81989; 1 ♂, 6 ♀, 7 nymphs, 1 adult: same data, except: serrinha, caldeirões em rocha, Água 7 station, 924 m, -8.5188, -37.2337, CEIOC 81991; 5 ♂, 4 ♀: same data, except: Buíque municipality, Serra do Catimbau, açudes, Água 9 station, 976 m, 14.III.2021. -8.5791, -37.2147, CEIOC 81990; 13 ♂, 15 ♀; same data, except: Buíque municipality, Fazenda Brejo de São José, brejo, bebedouro de cabras, Água 13 station, 673 m, -8.5247, -37.1968, CEIOC 82068. PI, 2018: 3 ♂: Caracol municipality, açude, Água 6 station, 512 m, 11.XII.2018. -9.2131, -43.4984. J.M.S. Rodrigues col., CEIOC 82037; 20 ♂, 24 ♀: Caracol municipality, PNSC, proximo a estrada, Terra 6 station, -9.2137, -43.4986. C.C. Gonçalves col., CEIOC 82065; 4 ♂: same data, except: J.M.S. Rodrigues col., CEIOC 82076; 4 ♂, 7 ♀: same data, except: próximo a estrada, Terra 8 station, light trap, 12.XII.2018. -9.2149, -43.5036. J.S. Prando col., CEIOC 82078; 2 ♂, 2 ♀: same data, except: CEIOC 82032; 3 ♂, same data, except: CEIOC 82038; 4 ♂, 11 ♀: same data, except: Jurema municipality, Sucumbido, Sede do Parque, Terra 11 station, 562 m, 13.XII.2018. -8.8597, -43.1817. J.M.S. Rodrigues col., CEIOC 82077. Same state, 2020: 1 ♂, 1 ♀: same data, except: Guaribas municipality, poças na estrada para Santa Luz, Água 5 station, 685 m, 14.II.2020. -9.376, -43.7914, CEIOC 81995; 2 ♂, 4 ♀: same data, except: Jurema municipality, Sucumbido, caldeirões, Água 7 station, 549 m, 15.II.2020. -8.8792, -43.1658. J.M.S. Rodrigues & I.R.S. Cordeiro cols., CEIOC 81986; 5 ♂; same data, except: área alagada perto da estrada, Água 8 station, 562 m, 15.II.2020. -8.8719, -43.1771, CEIOC 81984; 6 ♂, 5 ♀: Caracol municipality,PNSC, Povoado Sobrado, alagado em área de pasto, Água 11 station, 499 m, 16.II.2020. -9.2111, -43.5202, CEIOC 81988; 3 ♂, 7 ♀: same data, except: entrada da trilha Cores da Caatinga, Terra 3 station, light trap, 13.II.2020. -9.2135, -43.4665. J.M.S. Rodrigues col., CEIOC 81994; 1 ♂, 2 ♀: same data, except: cruzamento entre as estradas Santa Luz e Viana, Terra 8 station, light trap, 14.II.2020. -9.3845, -43.8064. J.S. Prando col., CEIOC 82028; 3 ♂, 2 ♀: same data, except: A.A. Alves col., CEIOC 82040; 2 ♂: same data, except: São Bráz municipality, estrada do Parque, Terra 11 station, light trap, 15.II.2020, -8.8451, -43.1847. J.M.S. Rodrigues col., CEIOC 81987; 1 ♂: same data, except: CEIOC 82026; 2 ♂, 1 ♀: same data, except: estrada do Parque, próximo a clareira de gado, Terra 14 station, light trap, 16.II.2020. -9.212, -43.5195, CEIOC 81992. !SE, 2018: 1 ♂, 11 ♀, 4 nymphs: Canhoba municipality, açude, SE1 station, 07.VII.2018. -10.1287, -36.9698. C.F.B. Floriano, J.M.S. Rodrigues & J.F. Barbosa cols., CEIOC 81612; 3 ♂: same data, except: Estância municipality, RBSI, Complexo de Dunas da Praia do Saco, REBIOSI17 station, 05.V.2018. -11.4190, -37.3223. C.F.B. Floriano, J.M.S. Rodrigues & O.M. Magalhães cols., CEIOC 81619; 3 ♂, 1 ♀: same data, except: CEIOC 81617. Same state, 2019: 7 ♂, 3 ♀: same data, except: Canindé de São Francisco municipality, açude, ALSE22 station, 301 m, 26.V.2019. -9.8549, -37.9391. J.M.S. Rodrigues, W. Souza & F.F.F. Moreira cols., CEIOC 82074; 7 ♂, 5 ♀: same data, except: área alagada, Riacho do Boqueirão, ALSE23 station, 280 m, -9.9096, -37.8837, CEIOC 82067.

#### ﻿ Buenoa unguis

Taxon classificationAnimalia

Truxal, 1953

8C03B9AC-F6C2-5C56-9B27-E17206FDBB57

Fig. 10L

Buenoa unguis Truxal, 1953: 1476–1479, fig. 78 [original description].

##### Diagnosis.

Medium to large species, male length 5.9–7.1 mm. Synthlipsis narrow, 1/6 to 1/5 of vertex width. Tylus flat-sulcate. Labial prong distinctly shorter than third labial article, originating proximally on article. Fore femur apex narrowed, without stridulatory area. Fore tarsus robust and dilated, with orthogonal-angulated claws.

##### Distribution.

Argentina (Truxal 1953). Bolivia (Truxal 1953). Brazil: AL [new record], AM (Rico et al. 2010; Barbosa and Nessimian 2013a), CE (Truxal 1953; Barbosa et al. 2010c; this work), MG (Truxal 1953; Nieser and Melo 1997; Melo and Nieser 2004; Barbosa et al. 2010c), PA (Truxal 1953; Andrade 1992; Barbosa and Nessimian 2013a), PB (Truxal 1953), PE (Truxal 1953; this work), PI (Takiya et al. 2016; this work), RJ (Truxal 1953; Barbosa et al. 2010c), RN (Truxal 1953), SE [new record], SP (Castanhole et al. 2013; Pereira et al. 2015a), TO (Truxal 1957). Paraguay (Truxal 1953). Peru (Truxal 1953). Venezuela (Herrera 2005).

##### Material examined.

Brazil, !AL, 2018: 2 ♂, 2 ♀: Piaçabuçu municipality, APAP, APAP4 station, 02.V.2018. -10.3252, -36.3506. C.F.B. Floriano, J.M.S. Rodrigues & O.M. Magalhães cols., CEIOC 81687. Same state, 2019: 4 ♂: same data, except: Marimbondo municipality, Flexeiras, açude, ALSE11 station, 122 m, -9.5074, -36.2340. J.M.S. Rodrigues, W. Souza & F.F.F. Moreira col., CEIOC 82047. CE, 2019: 3 ♂: Aiuaba municipality, EEA, Sítio do Martins, casa da gameleira, Água 1 station, 532 m, 09.IV.2019. -6.6992, -40.2908. J.M.S. Rodrigues col., CEIOC 81945; 1 ♂: same data, except: Sítio Volta de Cima, Sede do ICMBIO, poço, Água 6 station, 455 m, 10.IV.2019. -6.6019, -40.1248, CEIOC 82010; 9 ♂, 5 ♀: same data, except: Vaginha da Gameleira, açude, Água 9 station, 510 m, 12.IV.2019, -6.6866, -40.2625, CEIOC 81953; 1 ♂: same data, except: açude do Letreiro, Água 12 station, 475 m, 13.IV.2019. -6.6163, -40.1545, CZMA; 1 ♂, 2 ♀: same data, except: Sítio Volta de Baixo, Terra 12 station, light trap, 441 m, 14.IV.2019. -6.6254, -40.1336, CEIOC 81950. Same state, 2021: 1 ♂, 7 ♀: same data, except: Sítio Jatobá, açude, Água 1 station, 571 m, 06.VI.2021. -6.7351, -40.2424. J.M.S. Rodrigues col., CZMA; 1 ♂, 7 ♀: same data, except: Sítio Volta de Cima, lago, Água 4 station, 434 m, 05.VI.2021. -6.6019, -40.1246, CZMA; 10 ♂, 4 ♀: same data, except: brejo, Água 6 station, 570 m, -6.7263, -40.2229. J.M.S. Rodrigues & F.F.F Moreira cols., CEIOC 81954; 8 ♂, 12 ♀: same data, except: braço de rio, açude, Água 11 station, 513 m, 07.VI.2021. -6.6997, -40.1917, CEIOC 81952; 2 ♂, 1 ♀: same data, except: brejo, Água 12 station, 584 m, -6.7384, -40.2475, CZMA; 1 ♂: same data, except: estrada, Terra 3 station, light trap, 505 m, -6.6974, -40.2819. C.C. Gonçalves col., CZMA; 1 ♂: same data, except: estrada, Terra 5 station, light trap, 493 m, 03.VI.2021. -6.7003, -40.1919. J.M.S. Rodrigues col., CZMA; 2 ♂, 4 ♀: same data, except: estrada prox. ao riacho, Terra 16 station, light trap, 498 m, 06.VI.2021. -6.6871, -40.2683, CEIOC 81951. PE, 2019: 3 ♂: Buíque municipality, PNCA, riacho salgado do início da trilha para o Sítio arqueológico Alcobaça, poças temporárias, Água 6 station, 670 m, 15.III.2019. -8.5261, -37.1938. H. Rodrigues & J.M.S. Rodrigues cols., CEIOC 82062; 1 ♂, 4 ♀: same data, except: Tupanatinga municipality, brejo, perto da sede, poça temporária, Fazenda Brejo de São José, Água 7 station, 699 m, 16.III.2019. -8.5372, -37.2200, CZMA; 3 ♂: same data, except: riacho temporário, Fazenda Juá, Água 12 station, 548 m, 17.III.2019. -8.4350, -37.3467, CEIOC 82055; 4 ♂, 2 ♀: same data, except: Igrejinha, Barragem de Valdira, 853 m, Água 16 station, -8.4902, -37.2465, CEIOC 82052; 1 ♂: same data, except: Chapadão, poça temporária, 957 m, Água 18 station, 20.III.2019. -8.5244, -37.2394, CZMA; 2 ♂: same data, except: J.M.S. Rodrigues col., CEIOC 82061; 2 ♂: same data, except: H. Rodrigues col., CEIOC 82060; 3 ♂, 8 ♀, 1 nymph: same data, except: Fazenda do brejo, Terra 7 station, light trap, 706 m, 16.III.2019. -8.5199, -37.2249, J.M.S. Rodrigues col., CEIOC 82053; 3 ♂: same data, except: C.C. Gonçalves col., CEIOC 82048; 4 ♂, 6 ♀: same data, except: H. Rodrigues col., CEIOC 82044; 2 ♂, 3 ♀: same data, except: estrada do gado, Terra 14 station, light trap, 663 m, 19.III.2019. -8.4866, -37.3404. H. Rodrigues col., CZMA; 14 ♂, 32 ♀: same data, except: J.M.S. Rodrigues col., CEIOC 82056; 2 ♂: same data, except: Fazenda Laranjeiras, Terra 18 station, light trap, 610 m, 20.III.2019. -8.5244, -37.2394, CEIOC 82051. Same state, 2021: 2 ♂, 1 ♀: same data, except: Tupanatinga municipality, Barro branco, Comunidade Muquém, açudes, Água 1 station, 824 m, 10.III.2021. -8.5001, -37.3113. J.M.S. Rodrigues & R. Jordão cols., CEIOC 81947; 2 ♂, 3 ♀: same data, except: poça ao lado da estrada, Água 2 station, 904 m, -8.4982, -37.2852, CEIOC 81948; 1 ♂, 1 ♀: same data, except: Chapadão, açude, Água 3 station, 940 m, 12.III.2021. -8.5244, -37.2394, CZMA; 2 ♂, 4 nymphs: same data, except: caldeirões em rocha, ao lado de estrada de chão perto da Igrejinha, Água 6 station, 13.III.2021. -8.5024, -37.2543, CZMA; 1 ♂: same data, except: Serrinha, caldeirões em rocha, Água 7 station, 924 m, -8.5188, -37.2337, CZMA; 5 ♂, 4 ♀: same data, except: Buíque municipality, Serra do Catimbau, açudes, Água 9 station, 976 m, 14.III.2021. -8.5791, -37.2147, CEIOC 81949; 3 ♂, 3 ♀: same data, except: Fazenda Brejo São José, brejo, bebedouro de cabras, Água 13 station, 673 m, 16.III.2021. -8.5247, -37.1968, CZMA. PI, 2018: 3 ♂: PNSC, açude, Água 6 station, 512 m, 11.XII.2018. -9.213083, -43.498444, CEIOC 82063; 5 ♂, 7 nymphs: same data, except: Guaribas municipality, açude, Água 10 station, 640 m, 14.XII.2018. -9.364889, -43.754583, CEIOC 82049; 1 ♂, 2 ♀: same data, except: Caracol municipality, açude, Água 12 station, 498 m, 14.XII.2018. 9.2109, -43.5201, CEIOC 82050; 1 ♂, 1 ♀: Caracol municipality, PNSC, entre o mirante “Janela do Sertão” e o cemitério, Terra 2 station, light trap, 566 m, 10.XII.2018. -9.2188, -43.4902. J.S. Prando col., CEIOC 82046; 16 ♂: Caracol municipality, PNSC, proximo á estrada, Terra 6 station, light trap, 11.XII.2018. -9.2137, -43.4986. J.M.S. Rodrigues & O.M. Magalhães cols., CEIOC 82059; 1 ♂, 2 ♀: same data, except: J.S. Prando col., CZMA; 57 ♂, 110 ♀: same data, except: C.C. Gonçalves col., CEIOC 82045; 23 ♂: same data, except: próximo a estrada, Terra 8 station, light trap, 12.XII.2018. -9.2149, -43.5036. C.C. Gonçalves col., CEIOC 82058; 4 ♂: same data, except: J.M.S Rodrigues col., CZMA; 1 ♂: same data, except: Jurema municipality, casa do sucumbido, Sede do Parque, Terra 11 station, 562 m, 13.XII.2018. -8.8597, -43.1817, CEIOC 82054; 1 ♂, 1 ♀: same data, except: ao lado da estrada, em rio seco, Terra 15 station, light trap, 15.II.2018. -9.2137, -43.5000. J.M.S. Rodrigues & O.M. Magalhães cols., CZMA. same state, 2020: 1 ♂, 1 ♀: Guaribas municipality, PNSC, poças na estrada para Santa Luz, Água 5 station, 685 m, 14.II.2020. -9.376, -43.7914. J.M.S. Rodrigues & I.R.S. Cordeiro cols., CZMA; 2 ♂: same data, except: Jurema municipality, Sucumbido, caldeirões, Água 7 station, 549 m, 15.II.2020. -8.8792, -43.1658, CEIOC 82009; 3 ♂, 4 ♀: same data, except: área alagada perto da estrada, Água 8 station, 562 m, -8.8719, -43.1771, CEIOC 81946; 3 ♂, 16 ♀: same data, except Caracol municipality, povoado Sobrado, alagado em área de pasto, Água 11 station, 499 m, 16.II.2020. -9.2111, -43.5202, CZMA; 2 ♂, 7 ♀: same data, except: Guaribas municipality, Brejão, açude, Água 12 station, 640 m, 18.II.2020. -9.3650, -43.7544, CZMA; 12 ♂, 18 ♀: same data, except: Bom Jesus municipality, Viana, poças em estrada de terra, Água 13 station, 396 m, -9.4484, -43.0743, CEIOC 82008; 1 ♂: same data, except: São Bráz municipality, estrada do Parque, Terra 11 station, light trap, 15.II.2020. -8.8451, -43.1847. J.M.S. Rodrigues col., CZMA; 1 ♂, 1 ♀: same data, except: Caracol municipality, estrada do parque, próximo a clareira de gado, Terra 14 station, light trap, 16.II.2020. -9.212, -43.5195. J.S. Prando col., CZMA. !SE, 2018: 3 ♂, 20 ♀: Japaratuba municipality, Estrada BR-101, poça, REBIOSI8 station, 03.V.2018. -10.5476, -36.9698. C.F.B. Floriano, J.M.S. Rodrigues & O.M. Magalhães cols., CEIOC 81623. Same state, 2019: 6 ♂, 13 ♀: Canindé de São Francisco municipality, açude, ALSE22 station, 301 m, 26.V.2019. -9.8549, -37.9391. J.M.S. Rodrigues, W. Souza & F.F.F. Moreira cols., CEIOC 82057.

**Subfamily Notonectinae**

**Tribe Notonectini**

***Notonecta* Linnaeus, 1758**

#### ﻿ Notonecta (Paranecta) disturbata

Taxon classificationAnimalia

Hungerford, 1926

A9AA80B8-4FB7-5DC8-A881-F212B1C92E3B

Fig. 10M

Notonecta disturbata Hungerford, 1926: 13, 14, fig. 2 [original description].
Notonecta (Paranecta) disturbata ; Hutchinson (1929): 363 [subgenus placement].

##### Diagnosis.

Medium-sized, ~ 8 mm long. Vertex/synthlipsis ratio ~ 2.7. Lateral margins of pronotum straight and divergent. Genital capsule with two caudoventral protuberances; apex of paramere not bifurcated (Fig. 8A).

##### Taxonomic notes.

It is most similar to *N.pulchra* Hungerford, 1926, which has the same size. Examination of the male genital capsule is needed to ensure correct identification in most species of this genus, and *N.disturbata* is no exception. In this species, the genital capsule has two caudoventral protuberances, and dorsal portion is acute (Fig. 8A), opposing to *N.pulchra*, which has only one protuberance and the dorsal portion of the genital capsule rounded (Fig. 8B).

##### Distribution.

Argentina (Bachmann 1963). Brazil: AL [new record], CE [new record], ES (Bachmann 1963), GO (Truxal 1957), MG (Melo and Nieser 2004; Barbosa and Nessimian 2013b), MT (Nieser 1970), PA (Barbosa and Nessimian 2013b; Nobre et al. 2019), PE [new record], PI (Barbosa and Nessimian 2013b; Takiya et al. 2016), RJ (Hungerford 1926, 1933; Ribeiro et al. 1998, 2010; Barbosa and Nessimian 2013b), RS (Bachmann 1963; Barbosa and Nessimian 2013b), SE [new record], SP (Barbosa and Nessimian 2013b), TO (Truxal 1957). Paraguay (Hungerford 1933).

##### Material examined.

Brazil, !AL, 2018: 1 ♂, 1 ♀: Quebrângulo municipality, RBPT, Sítio Juliana, brejo, PPT4 station, 04.VII.2018. -9.2608, -36.4157. C.F.B. Floriano, J.M.S. Rodrigues & J.F. Barbosa cols., CEIOC 81679. Same state, 2019: 1 ♂, 2 ♀: same data, except: Minador do Negrão municipality, açude, ALSE13 station, 253 m, 23.V.2019. -9.3679, -36.8425. J.M.S. Rodrigues, W. Souza & F.F.F. Moreira cols., CEIOC 82013; 3 ♂, 2 adults: same data, except: Maravilha municipality, rodovia BR-316, açude, ALSE15, 294 m, 24.V.2019, CEIOC 82014. !CE, 2019: 2 ♂: Aiuaba municipality, EEA, área alagada ao lado da estrada para a casa do cajueiro, Água 4 station, 508 m, 10.IV.2019. -6.6923, -40.1873. J.M.S. Rodrigues col., CEIOC 81941; 1 ♂: same data, except: sítio Boa Vista, área alagada, Água 7 station, 570 m, 11.IV.2019. -6.7255, -40.2226, CEIOC 81943. Same state, 2021: 1 ♂, 2 ♀: same data, except: brejo, Água 6 station, 570 m, 05.VI.2021. -6.7263, -40.2229. J.M.S. Rodrigues & F.F.F Moreira cols., CEIOC 81944. !PE, 2019: 1 ♂: Tupanatinga municipality, PNCA, Fazenda Brejo de São José, poça temporária, Água 8 station, 700 m, 16.III.2019. -8.506, -37.2237. H. Rodrigues & J.M.S. Rodrigues cols., CEIOC 82015; 2 ♂, 1 ♀: same data, except: Chapadão, poça temporária, Água 18 station, 957 m, 20.III.2019. -8.5244, -37.2394, CEIOC 81942. !SE, 2018: 1 ♂: Areia Branca municipality, PNSI, Riacho Negro, PNSI2 station, 08.VII.2018. -10.7475, -37.3402. C.F.B. Floriano, J.F. Barbosa J.M.S Rodrigues cols., CEIOC 81674; 2 ♂, 2 ♀, 1 nymph: Lagarto municipality, Estrada SE-170, açude, 09.VII.2018. [-10.91319, -37.67136]. C.F.B. Floriano, J.M.S. Rodrigues & J.F. Barbosa cols., CEIOC 81661. Same state, 2019: 5 ♂, 3 ♀: same data, except: Canindé de São Francisco municipality, açude, ALSE22 station, 301 m, 26.V.2019. -9.8549, -37.9391. J.M.S. Rodrigues, W. Souza & F.F.F. Moreira cols., CEIOC 81940.

***Enitharoides* Brooks, 1953**

#### ﻿ Enitharoides brasiliensis

Taxon classificationAnimalia

(Spinola, 1837)

B6D3E2EF-4D8A-5C37-8CE4-3D5557DFC36B

Fig. 10N

Enithares brasiliensis Spinola, 1837: 60 [original description].
Notonecta grandis Germar in Herrich-Schäffer, 1849: 42, fig. 901 [synonym; Kirkaldy (1904): 101].
Bothronotus brasiliensis ; Fieber (1852): 472 [unnecessary generic replacement name].
Enithares (Enitharoides) brasiliensis ; Brooks (1953): 74, 75, figs A, D [subgenus placement].
Enitharoides brasiliensis ; Štys and Říha (1975): 166 [genus transfer].

##### Diagnosis.

Male length 15–16 mm. Wide rectangular punctuated area on scutellum. Middle femur with short and dark pubescence; anteapical protuberance weakly developed. Apex of metaxyphus variable, acute to spatulated.

##### Taxonomic notes.

This species shows some variation in general and hemelytral color pattern, from light-yellow to dark brown, with deep punctures on the scutellum. The amount of these punctures seems to follow the overall color of the specimens. It resembles *E.lanemeloi* Barbosa, Ribeiro & Nessimian, 2017, as both species show middle femur with short setae and poorly developed apical spur, but can be distinguished from it by the coarser aspect and different pattern of the scutellar punctures, the different pattern of the punctuated area on the scutellum, and by differences in paramere shape. Differences between *E.brasiliensis* and the other species occurring in the study area, *E.tricomerus*, are provided in the key.

##### Distribution.

Brazil: BA [new record], ES (Kirkaldy 1904), MG (Kirkaldy 1904; Nieser and Melo 1997; Goulart et al. 2002; Vianna and Melo 2003; Pelli et al. 2006; Souza et al. 2006; Barbosa et al. 2017), RJ (Henriques-Oliveira and Nessimian 2010; Ribeiro et al. 2010; Cordeiro and Moreira 2015; Barbosa et al. 2017), SP (Barbosa et al. 2017).

##### Material examined.

Brazil, !BA, 2021: 1 ♂, 2 adults, 1 nymph: Mucugê municipality, PNCD, rio próximo ao Mirante do Cachoeirão, Água 10 station, 1191 m, 19.V.2021. -12.8110, -41.4429. J.M.S. Rodrigues col., CEIOC 81956.

#### ﻿ Enitharoides tricomerus

Taxon classificationAnimalia

Barbosa, Ribeiro & Nessimian, 2017

1CF9F526-5B16-5AC9-BD98-7C90191F3413

Fig. 10O

Enitharoides tricomerus Barbosa, Ribeiro & Nessimian, 2017: 480, 482, figs 49–61 [original description].

##### Diagnosis.

Male length 15.3–16.8 mm. Deeply punctuated area widely distributed on scutellum. Middle femur with long pubescence and robust anteapical protuberance. Apex of metaxyphus triangular.

##### Taxonomic notes.

This species shows conspicuous long brown setae and a well-developed anteapical spur on the middle femur. These features are present in another species, *E.lucasduquei* Barbosa, Ribeiro & Nessimian, 2017, but the latter has a triangular-shaped spur on the middle femur and a different pattern of scutellar punctures. Also, the middle femur of *E.lucasduquei* bears shorter setae restricted to the apical half of the segment, and shows a less dense pubescence aspect.

##### Distribution.

Brazil: AL [new record], ES (Barbosa et al. 2017), MG (Barbosa et al. 2017).

##### Material examined.

Brazil, !AL, 2018: 1 ♂: Murici municipality, EEM, EEM4 station, 28.IV.2018. -9.2540, -35.8010. C.F.B. Floriano, J.M.S. Rodrigues & O.M. Magalhães cols., CEIOC 81659. Same state, 2019: 2 ♂, 1 ♀, 2 nymphs: same data, except: Vale do Socorro, riacho sem nome em área de dendê, ALSE6 station, 22.V.2019. -9.2386, -35.8649. J.M.S. Rodrigues, W. Souza & F.F.F. Moreira cols., CEIOC 82016; 4 ♂, 3 ♀, 1 nymph: same data, except: Fazenda Bananeira, riacho, ALSE9 station, -9.2193, -35.8789, CEIOC 82017.

**Tribe Nychiini**

***Martarega* White, 1879**

#### ﻿ Martarega bentoi

Taxon classificationAnimalia

Truxal, 1949

86D1416C-3DEC-55DA-B39F-1F612FF641E2

Fig. 10P, Q

Martarega bentoi Truxal, 1949: 22, 23, figs 7, 8 [original description].

##### Diagnosis.

Male length 5.2–5.3 mm. Ocular commissure > 1/3 of eye width. Scutellum with hyaline apex; ventral surface of middle trochanter with a group of ensiform bristles. Hemelytra of brachypterous specimens with medial longitudinal hyaline stripe wide, tapering from base of hemelytra up to coastal margin in membrane suture; wide range of hemelytral process stripe, 25% up to 40% of hemelytral length; hemelytral process shorter than membrane length (Fig. 9A).

**Figure 11.**
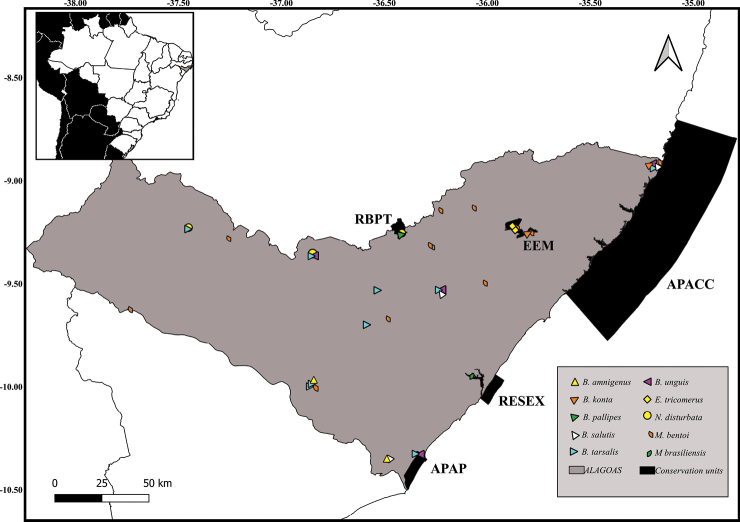
New records of Notonectidae species in Alagoas State, Brazil.

**Figure 12.**
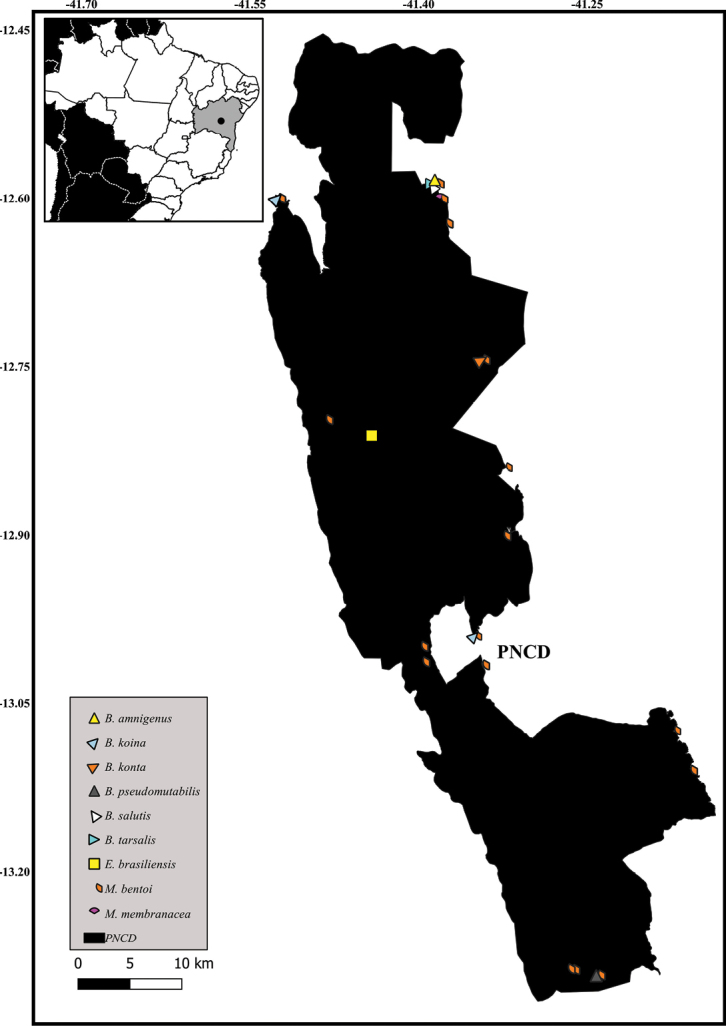
New records of Notonectidae species in Parque Nacional da Chapada Diamantina, Bahia State, Brazil.

**Figure 13.**
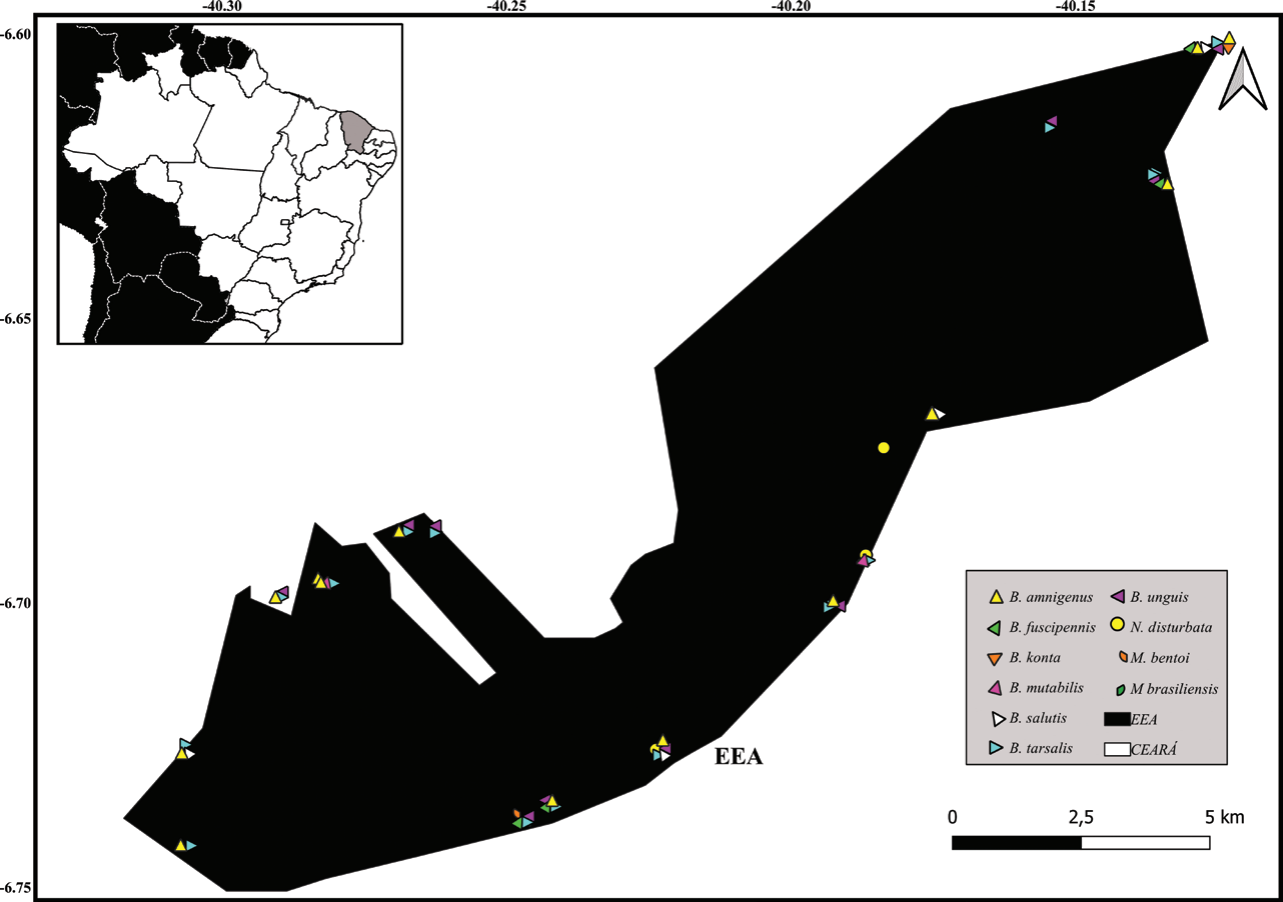
New records of Notonectidae species in Estação Ecológica de Aiuaba, Ceará State, Brazil.

**Figure 14.**
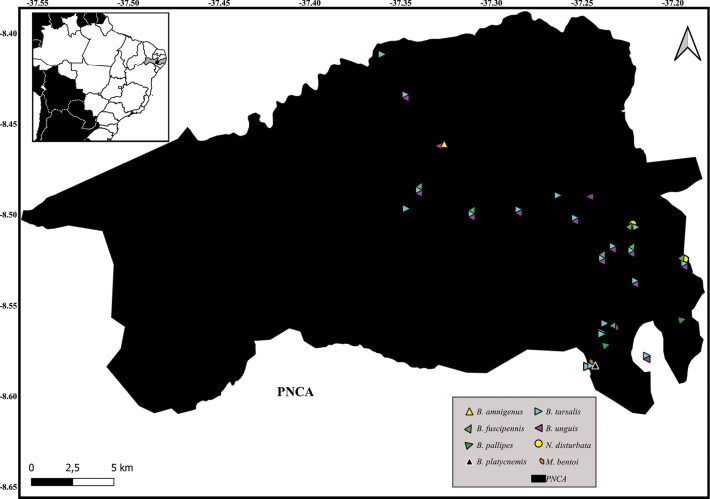
New records of Notonectidae species in Parque Nacional do Catimbau, Pernambuco State, Brazil.

**Figure 15.**
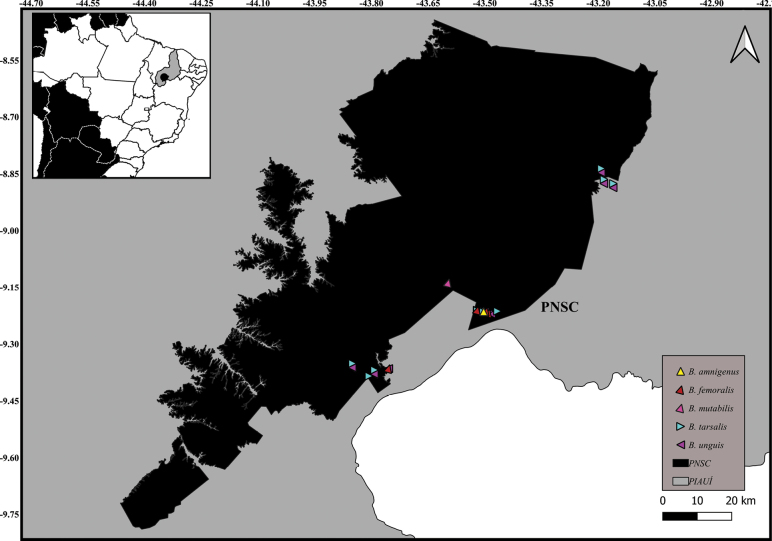
New records of Notonectidae species in Parque Nacional da Serra das Confusões Piauí State, Brazil.

**Figure 16.**
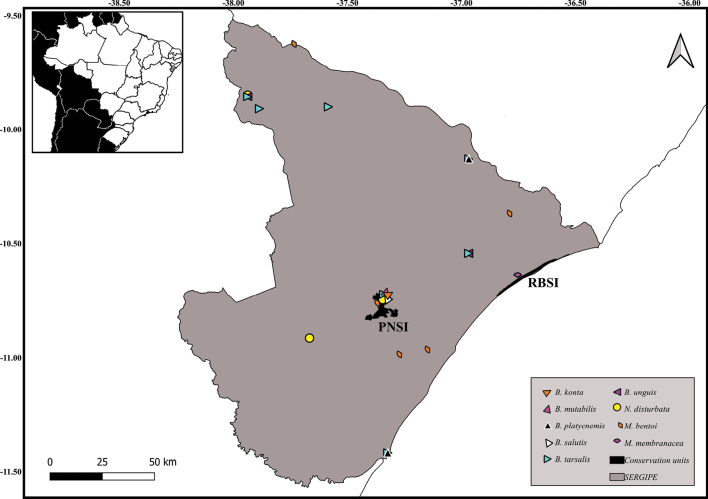
New records of Notonectidae species in Sergipe State, Brazil.

**Figure 17.**
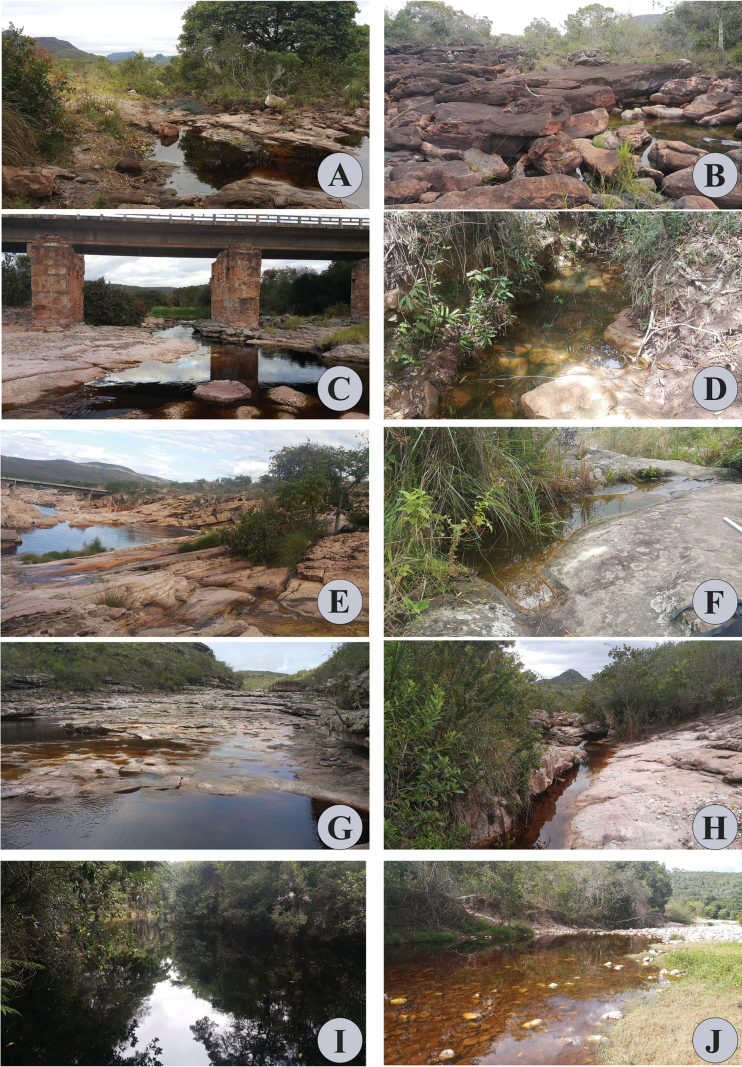
Aspects of some localities surveyed in Parque Nacional da Chapada Diamantina, Bahia State, Brazil, 2018 **A** Água 1 station **B** Água 2 station **C** Água 3 station **D** Água 8 station **E** Água 6 station **F** Água 4 station **G** Água 7 station **H** Água 5 station **I** Água 11 station **J** Água 9 station.

**Figure 18.**
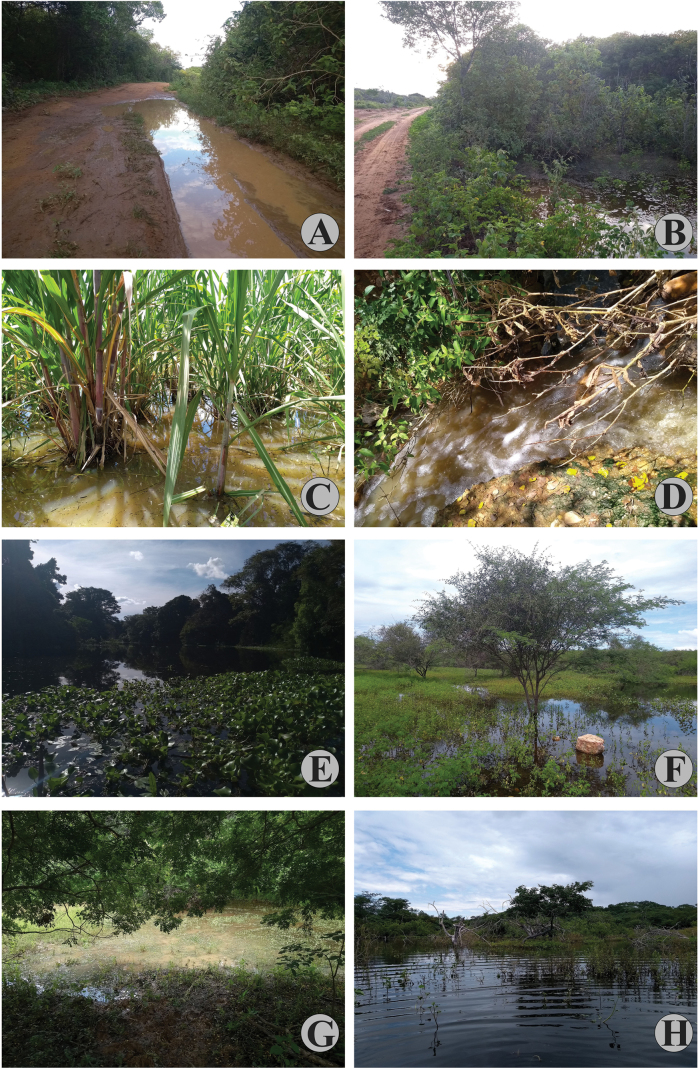
Aspects of some localities surveyed in Estação Ecológica de Aiuaba, Ceará State, Brazil, 2019 **A** Água 2 station **B** Água 3 station **C** Água 4 station **D** Água 5 station **E** Água 6 station **F** Água 7 station **G** Água 10 station **H** Água 13 station.

**Figure 19.**
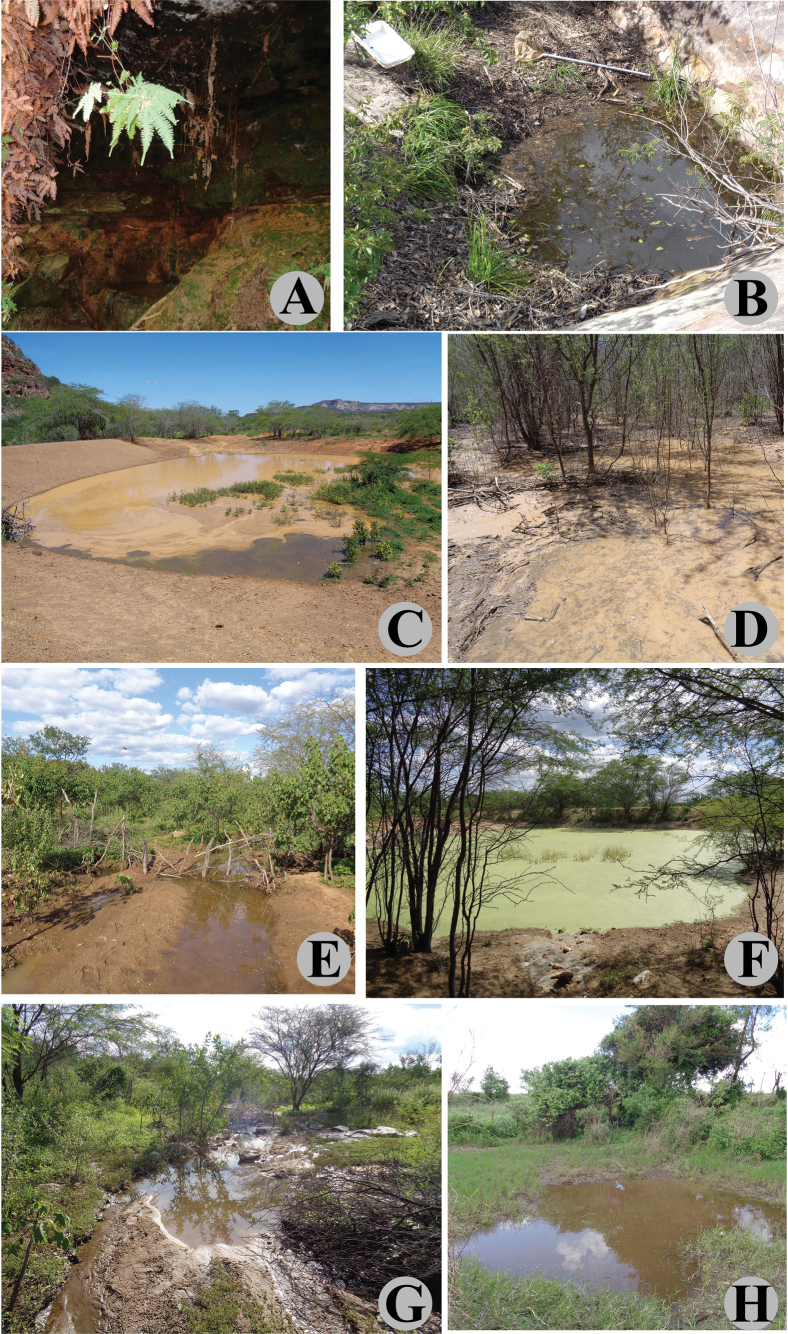
Aspects of some localities surveyed in Parque Nacional do Catimbau, Pernambuco State, Brazil, 2019 **A** Água 2 station **B** Água 4 station **C** Água 8 station **D** Água 6 station **E** Água 12 station **F** Água 11 station **G** Água 12 station **H** Água 13 station.

**Figure 20.**
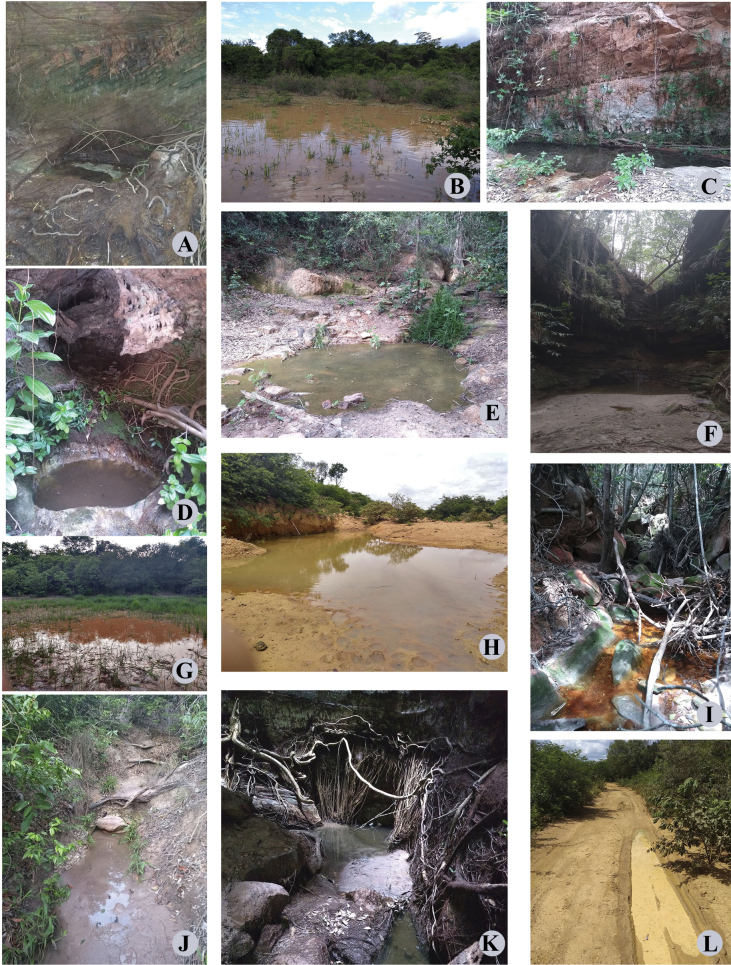
Aspects of some localities surveyed in Parque Nacional da Serra das Confusões, Piauí State, Brazil, 2018 **A** Água 4 station **B** Água 6 station **C** Água 3 station **D** Água 4 station **E** Água 13 station **F** Água 8 station **G** Água 12 station **H** Água 9 station **I** Água 7 station **J** Água 2 station **K** Água 5 station **L** Água 11 station.

##### Taxonomic notes.

*Martaregabentoi* is a medium-sized species that is very common and widespread in Brazil. It is very similar to *M.uruguayensis* (Berg, 1883), but can be distinguished from it after examination of the hyaline band on the hemelytra of coleopteroid specimens (Fig. 9A), or the different position of the ensiform bristles on the middle trochanter. Also, it differs from *Martaregabrasiliensis* and *M.membranacea*, the other two species found in this survey, by the combination of a scutellum with a hyaline apex and the middle trochanter with ensiform bristles.

##### Distribution.

Argentina (Mazzucconi 2008). Brazil: AL [new record], BA (Moreira et al. 2016; this work), CE (Takiya et al. 2016; this work), GO (Barbosa and Dias-Silva 2017), MG (Barbosa and Rodrigues 2013; Gutiérrez et al. 2017b; Novais et al. 2017), MT (Barbosa and Giehl 2014), PE (Truxal 1949, this work), PI (Barbosa and Rodrigues 2013), RJ (Cordeiro and Moreira 2015), SE [new record].

##### Material examined.

Brazil, !AL, 2018: 1 ♂: Murici municipality, EEM, EEM5 station 28.IV.2018. -9.2540, -35.8010. C.F.B. Floriano, J.M.S. Rodrigues & O.M. Magalhães cols., CEIOC 81654; 1 ♂, 1 ♀: Maragogi municipality, APACC, APACC2 station, 29.IV.2018. -8.9220, -35.1795, CEIOC 81656; 10 ♂, 4 ♀, 5 nymphs: same data, except: APACC3 station, -8.9307, -35.2105, CEIOC 81678; 8 ♂, 8 ♀, 5 nymphs: Coité do Nóia municipality, Taquarana, queda d’água, AL4 station, 06.VII.2018. -9.6723, -36.4987. C.F.B. Floriano, J.M.S. Rodrigues & J.F. Barbosa cols., CEIOC 81657; 1 ♂: Campo Grande municipality, açude, AL7 station, 07.VII.2018. -9.9559, -36.8373, CEIOC 81668. Same state, 2019: 4 ♂, 4 ♀, 9 nymphs: same data, except: União dos Palmares, municipality, Rio Mundaú, ALSE1 station, 21.V.2019. -9.1342, -36.0809. J.M.S. Rodrigues, H. Rodrigues, W. Souza, F.F.F. Moreira cols., CEIOC 82083; 4 ♂, 4 ♀: same data, except: Santana do Mundaú municipality, rio na estrada AL-205, ALSE2 station, -9.1464, -36.2430, CEIOC 82081; 16 ♂, 6 ♀, 1 nymph: same data, except: Viçosa municipality, Fazenda da Baixa Funda, riacho, ALSE3 station, -9,3243, -36.2830, CEIOC 82085; 7 ♂, 4 ♀, 2 nymphs: same data, except: Murici municipality, EEM, Vale do Socorro, Cachoeira do Socorro, ALSE5 station, 361 m, 22.V.2019. -9.236, -35.8609, CEIOC 82084; BA, 2018: 4 ♂, 3 nymphs: Mucugê municipality, PNCD, Água 2 station, 19.VIII.2018. -13.0004, -41.3982. J.M.S. Rodrigues & F.F.F Moreira cols., CZMA; 8 ♂, 2 ♀, 40 nymphs: same data, except: Água 3 station, -12.9908, -41.3507, CEIOC 81931; 4 ♂, 8 ♀, 12 nymphs: same data, except: Andaraí municipality, Água 5 station, 20.VIII.2018. -12.9002, -41.3249, CZMA; 6 ♂, 2 ♀, 5 nymphs: same data, except: Água 6 station, -12.8401, -41.3235, CEIOC 81928; 1 ♀, 4 nymphs: same data, except: Mucugê municipality, Água 7 station, 21.VIII.2018. -13.0174, -41.3441, CZMA; 1 ♂, 2 ♀, 4 nymphs: same data, except: Lençóis municipality, Água 9 station, 22.VIII.2018. -12.5865, -41.3822, CZMA; 1 ♂, 2 ♀: same data, except: Água 10 station, -12.6008, -41.3811, CZMA; 3 ♂, 4 nymphs: same data, except: Mucugê municipality, Água 11 station, 23.VIII.2018. -13.2932, -41.2414, CZMA; 1 ♂, 5 ♀, 1 nymph: same data, except: Água 12 station, -13.2886, -41.2646, CEIOC 81925. Same state, 2021: 6 ♂, 6 ♀, 1 nymph: same data, except: Palmeiras municipality, Rio Preto, Água 1 station, 820 m, 05.V.2021. -12.6036, -41.5250. J.M.S. Rodrigues & J.F. Barbosa cols., CEIOC 81927; 2 ♂, 3 ♀, 5 nymphs: same data, except: Mucugê municipality, Cachoeira Véu da Noiva, Água 2 station, 776 m, 06.V.2021. -13.2881, -41.2683, CZMA; 19 ♂, 6 ♀, 1 nymph: same data, except: Itaeté municipality, Rio Timbó, Água 3 station, 428 m, 07.V.2021. -12.6036, -41.5250, CEIOC 81930; 1 ♂ (macropterous): same data, except: CEIOC 81926; 3 ♂, 4 ♀: same data, except: Rio Samina, Água 4 station, 413 m, -13.1105, -41.1587, CZMA; 2 ♂, 3 ♀: same data, except: Andaraí municipality, Rio Garapa, Água 5 station, 330 m, 08.V.2021. -12.7456, -41.3454, CZMA; 6 ♂, 2 ♀, 2 nymphs: same data, except: Lençóis municipality, Rio Capivara, Água 8 station, 405 m, 09.V.2021. -12.6232, -41.3763, CZMA; 1 ♂ 1 ♀: same data, except: Mucugê municipality, Rio Preto, Água 9 station, 1249 m, 10.V.2021. -12.7978, -41.4835, CZMA; 1 ♂ (macropterous): same data, except: Cachoeira da Moça Loira, Água 11 station, 724 m, -12.7978, -41.4835, CEIOC 81929; 8 ♂, 3 ♀: same data, except: CEIOC 81924; 2 ♂, 3 ♀: same data, except: Mucugê, Rio Una, Água 12 station, 11.V.2021. -13.2921, -41.2524, CEIOC 82020. CE, 2021: 1 ♂, 1 ♀ (both macropterous), 1 nymph: Aiuaba municipality, EEA, brejo, Água 2 station, 489 m, 04.VI.2021. -6.6850, -40.1841, CEIOC 82018. PE, 2019: 15 ♂, 16 ♀: Tupanatinga municipality, PNCA, Riacho de Leís, poças temporárias ao longo do leito, Água 14 station, 750 m, 18.III.2019. -8.5829, -37.2445. J.M.S. Rodrigues & H. Rodrigues cols., CEIOC 82019. !SE, 2018: 12 ♂, 2 ♀: Areia Branca municipality, PNSI, Riacho Negro, PNSI2 station, 08.VII.2018. -10.7475, -37.3402, C.F.B. Floriano, J.F. Barbosa, J.M.S. Rodrigues cols., CEIOC 81655; 14 ♂, 4 ♀: same data, except: Riacho Vermelho, PNSI1 station, -10.7387, -37.33547, CEIOC 81675; 4 ♂, 5 ♀, 12 nymphs: Japoatã municipality, Estrada SE-335, RebioSI7 station, 03.V.2018. -10.3679, -36.8074. C.F.B. Floriano, J.M.S. Rodrigues & O.M. Magalhães cols., CEIOC 81667; 3 ♂, 2 ♀, 3 nymphs: São Cristóvão municipality, Rio Pitanga, RebioSI11 station, 04.V.2018. -10.9633, -37.1663, CEIOC 81669; 11 ♂, 8 ♀, 7 nymphs: same data, except: Estrada Rita Cacete, RebioSI12 station, -10.9851, -37.2868, CEIOC 81677.

#### ﻿ Martarega brasiliensis

Taxon classificationAnimalia

Truxal, 1949

0EE1C5BC-0FBD-55FB-988E-0E1E53317AEA

Fig. 10R

Martarega brasiliensis Truxal, 1949: 16, figs 3, 4 [original description].

##### Diagnosis.

Medium-sized species, male length 5.2–5.4 mm. Ocular commissure 1/2 of eye width. Ventral surface of third labial article pubescent. Scutellum with hyaline apex. Fore femur with a short spiniform seta. Ventral surface of middle trochanter with a patch of thin setae. Hemelytra of brachypterous specimens with medial longitudinal hyaline stripe narrow, slightly tapering from base of hemelytra up to membrane suture; hemelytral process stripe short, 20–30% of hemelytral length; hemelytral process slightly shorter than membrane length (Fig. 9B).

##### Taxonomic notes.

*Martaregabrasiliensis* is slightly larger than *M.nessimiani* Barbosa & Rodrigues, 2013, a species with which it shares some similarities. *Martaregabrasiliensis* has some distinctive features, like the pubescent labial surface and differences in the width of the hyaline band of the hemelytra.

##### Distribution.

Brazil: AL [new record], CE (Truxal 1949; this work), GO (Barbosa and Dias-Silva 2017), MS (Floriano et al. 2013), MT (Barbosa and Giehl 2014), PA (Nieser 1970; Barbosa et al. 2010b, 2012), PE (Truxal 1949), RJ (Ribeiro et al. 2010), RR (Barbosa et al. 2012; Barbosa and Rodrigues 2013), SP (Castanhole et al. 2013; Pereira et al. 2015a, 2015b). Colombia (Padilla-Gil 2014). Peru (Truxal 1949). Suriname (Nieser 1968).

##### Material examined.

Brazil, !AL, 2018: 2 ♂, 16 ♀, 4 nymphs: Jequiá da Praia municipality, RESEX, Mutuca, RJ3 station, 30.IV.2018. -9.9446, -36.0846. C.F.B. Floriano, J.M.S. Rodrigues & O.M. Magalhães cols., CEIOC 81663; 2 ♂, 6 ♀: same data, except: RJ4 station, -9.9502, -36.0859, CEIOC 81660. CE, 2019: 1 ♂: Aiuaba municipality, EEA, Sítio Volta de Cima, sede do ICMBIO, poço, Água 6 station, 455 m, 10.IV.2019. -6.6019, -40.1248. J.M.S. Rodrigues col., CEIOC 81932; 1 ♂: same data, except: Sítio Volta de cima, sede do ICMBIO, Terra 4 station, light trap, 460 m, -6.6025, -40.1286. J.S. Prando col., CZMA; 1 ♂: same data, except: Sítio Boa Vista, Terra 6 station, Light trap, 583 m, 13.IV.2019. -6.6025, -40.1286. V. Quintas col., CEIOC 81957; 1 ♂: same data, except: J.S. Prando col., CEIOC 81933.

#### ﻿ Martarega membranacea

Taxon classificationAnimalia

White, 1879

9C28E17B-49E9-5FA4-A837-C9031473123E

Fig. 10S

Martarega membranacea White 1879: 272 [original description].

##### Diagnosis.

Small species, male length 4.1–4.2 mm. Ocular commissure longer than 1/2 of eye width. Scutellum with 1/2 of its length hyaline. Ventral surface of middle trochanter smooth. Hemelytra of brachypterous specimens with claval commissure expanded, hyaline, reaching the anterior margin of membrane; medial longitudinal hyaline stripe very wide, sinuous, with same width from base of hemelytra up to coastal margin in membrane suture; hemelytral process stripe large, 20–30% of hemelytral length; hemelytral process slightly longer than membrane length (Fig. 9C)

##### Taxonomic notes.

This species can be distinguished from *M.chinai* Hynes, 1948, the most similar congener, by the longer posterior hemelytral process and by the absence of a pubescent nodule on the middle trochanter.

##### Distribution.

Argentina (López-Ruf et al. 2003). Bolivia (Truxal 1949). Brazil: AM (White 1879; Truxal 1949; Nieser 1970; Pereira and Melo 2007; Barbosa et al. 2012; Barbosa and Rodrigues 2013), BA [new record], GO (Nieser 1970; Barbosa and Dias-Silva 2017), MG (Melo and Nieser 2004; Barbosa and Rodrigues 2013), MS (Floriano et al. 2013), MT (Barbosa and Giehl 2014), PA (Truxal 1949; Nieser 1970; Andrade 1992; Barbosa et al. 2012), RJ (Ribeiro et al. 2010), RO (Truxal 1949), SE [new record], SP (Castanhole et al. 2013; Pereira et al. 2015a), TO (Truxal 1957). Colombia (Roback and Nieser 1974). Ecuador (Kirkaldy 1899c). Guyana (Truxal 1949). Suriname (Nieser 1968).

##### Material examined.

Brazil, !BA, 2018: 2 ♂, 11 ♀: Lençóis municipality, PNCD, Água 10 station, 22.VIII.2018. -12.6008, -41.3811, J.M.S. Rodrigues & F.F.F Moreira cols., CEIOC 81934. !SE, 2018: 3 ♂, 3 ♀, 2 nymphs: Pirambu municipality, RBSI, RebioSI5, 01.V.2018. -10.6515, -36.7571. C.F.B. Floriano, J.M.S. Rodrigues & O.M. Magalhães cols., CEIOC 81662.

## ﻿Discussion

### ﻿General distribution of represented species

In this survey, approximately 1400 specimens from six of the nine states in northeastern Brazil have been examined, representing 18 (ca 31%) of the 58 species of the Notonectidae recorded from Brazil (Moreira et al. 2011; Ribeiro et al. 2022). Among the studied species, six (35%) are endemic from the country and 15 (88%) are present only in South America (including Trinidad & Tobago), with the exceptions of *Buenoamutabilis*, *B.platycnemis*, and *B.pallipes*. This last species has a wide distribution, from Mexico to Paraguay, but had not been found in Brazil since the second half of the 20^th^ century (Barbosa and Nessimian 2013a). Among the backswimmer species occurring in Brazil, only 12 had been previously recorded from the northeastern region of the country, a number that here is elevated to 18. The species firstly recorded from the region are *Buenoafemoralis*, *B.fuscipennis*, *B.koina*, *B.konta*, *B.pallipes*, *Enitharoidesbrasiliensis*, *E.tricomerus*, and *Martaregamembranacea*. Our data show that the distribution range of *Buenoa* and *Enitharoides* species are wider and much more environmentally variable than what had been reported in the literature.

### ﻿Distribution of represented species in Brazilian biomes

Of all species collected, only *Buenoasalutis* is present in all Brazilian biomes. *Buenoaamnigenus*, *B.konta*, and *Notonectadisturbata* are second in numbers of biomes occupied, the first two lacking records from the Pampa, and the last one, from the Pantanal. Many species found in this survey (10 spp., 55%) are present in other three Brazilian biomes, as shown in Table 1. This reinforces the already observed similarity of the biota found in the Caatinga with that present in adjacent biomes (Santos et al. 2007; Takiya et al. 2016; Rodrigues et al. 2021).

**Table 1.** Distribution of Notonectidae species based on their records in States. Normal font: previous records; bold font: new records; *italic*: same states, different localities.

**Biome**		**Caatinga**									**Atlantic Forest**											**Pampas**
**State**		**AL**	**BA**	**CE**	**MA**	**PB**	**PE**	**PI**	**RN**	**SE**	**AL**	**ES**	**MG**	**MS**	**PE**	**PR**	**RJ**	**RS**	**SC**	**SE**	**SP**	**RS**
**Subfam.**	**Species**																					
**Anisopinae**	*B.amnigenus*	**X**	X	***X*** *X*	–	X	X	**X**	X	–	**X**	–	X	–	–	–	–	–	–	–	X	–
	*B.femoralis*	–	–	–	–	–	–	**X**	–	–	–	–	–	–	–	X	–	–	–	–	–	–
	*B.fuscipennis*	–	–	**X**	–	–	**X**	–	–	–	–	–	–	–	–	X	–	–	X	–	–	–
	*B.koina*	–	**X**	–	–	–	–	–	–	–	–	–	X	–	–	–	–	–	–	–	–	–
	*B.konta*	–	**X**	**X**	–	–	–	–	–	–	**X**	–	X	–	–	–	X	–	–	**X**	–	–
	*B.mutabilis*	–	–	**X**	–	–	–	X	–	–	–	–	X	–	–	–	–	–	–	**X**	–	–
	*B.pallipes*	–	X	–	–	–	**X**	–	–	–	**X**	–	–	–	–	–	–	–	–	–	–	–
	*B.platycnemis*	–	–	–	X	–	**X**	–	–	–	–	–	–	–	–	–	X	–	–	**X**	–	–
	*B.pseudomutabilis*	–	**X**	–	–	–	–	X	–	–	–	–	–	–	–	–	X	–	–	–	–	–
	*B.salutis*	–	**X**	***X*** *X*	–	X	–	X	–	–	**X**	–	X	X	–	X	X	–	–	**X**	X	X
	*B.tarsalis*	**X**	***X*** *X*	**X** *X*	–	X	***X*** *X*	***X*** *X*	X	–	**X**	–	X	–	X	–	X	–	–	**X**	–	–
	*B.unguis*	–	**X** *X*	**X** *X*	–	X	**X**	***X*** *X*	X	–	**X**	–	X	–	X	–	X	–	–	**X**	X	–
	*N.disturbata*	–	–	**X**	–	–	–	X	–	**X**	**X**	–	X	–	–	–	X	–	–	**X**	X	X
**Notonectinae**	*E.brasiliensis*	–	**X**	–	–	–	–	–	–	–	–	–	X	–	–	–	X	–	–	–	X	–
	*E.tricomerus*	–	–	–	–	–	–	–	–	–	**X**	X	X	–	–	–	–	–	–	–	–	–
	*M.bentoi*	**X**	**X**	**X** *X*	–	–	–	X	–	–	**X**	–	X	–	X	–	X	X	–	**X**	–	–
	*M.brasiliensis*	–	–	**X** *X*	–	–	–	–	–	–	**X**	–	–	X	–	–	X	–	–	–	X	–
	*M.membranacea*	–	**X**	–	–	–	–	–	–	–	–	–	–	X	–	–	X	–	–	**X**	–	–
**Biome**		**Amazon Forest**							**Cerrado Savannah**									**Pantanal**				
**State**		**AC**	**AP**	**AM**	**PA**	**MT**	**RO**	**RR**	**DF**	**GO**	**MA**	**MG**	**MS**	**MT**	**PI**	**SP**	**TO**	**MS**	**MT**			
**Subfam.**	**Species**																					
**Anisopinae**	*B.amnigenus*	–	–	X	X	–	–	–	–	X	–	–	–	–	**X**	X	–	X	X			
	*B.femoralis*	–	–	X	–	–	–	–	–	–	–	–	–	–	–	–	–	–	–			
	*B.fuscipennis*	–	–	–	X	–	–	–	–	–	–	–	–	–	–	–	–	–	X			
	*B.koina*	–	–	–	–	–	–	–	–	–	–	X	–	–	–	–	–	–	–			
	*B.konta*	–	–	–	X	–	–	–	–	X	–	X	–	–	–	–	–	X	–			
	*B.mutabilis*	–	–	–	–	–	–	–	–	X	–	X	–	–	–	–	–	–	–			
	*B.pallipes*	–	–	X	X	–	–	–	–	–	–	–	–	–	–	–	–	–	–			
	*B.platycnemis*	–	–	X	X	–	–	–	–	X	–	–	–	X	–	–	X	–	–			
	*B.pseudomutabilis*	–	–	–	–	–	–	–	–	X	–	–	–	–	–	–	–	–	–			
	*B.salutis*	–	–	X	X	–	–	X	–	–	–	X	–	–	–	–	X	X	–			
	*B.tarsalis*	–	–	X	X	–	–	–	–	X	–	X	–	–	**X**	–	–	–	–			
	*B.unguis*	–	–	X	X	–	–	–	–	–	–	X	–	–	**X**	X	–	–	–			
	*N.disturbata*	–	–	–	X	–	–	–	–	X	–	X	–	–	–	–	X	–	–			
	*E.brasiliensis*	–	–	–	–	–	–	–	–	–	–	X	–	–	–	–	–	–	–			
	*E.tricomerus*	–	–	–	–	–	–	–	–	–	–	–	–	–	–	–	–	–	–			
**Notonectinae**	*M.bentoi*	–	–	–	–	X	–	–	–	X	–	X	–	X	–	–	–	–	–			
	*M.brasiliensis*	–	–	–	X	–	–	X	–	X	–	–	–	X	–	X	–	–	–			
	*M.membranacea*	–	–	–	–	–	–	–	X	X	–	X	–	X	–	X	–	–	–			

### ﻿Distribution of represented species in the conservation units studied

The richest conservation unit was EEA (CE), with records of three genera and ten species, representing more than half (56%) of the surveyed diversity despite being the fifth in total area (MMA 2022). The least species-rich unit was RESEX, with only *M.brasiliensis*. The most frequent and abundant species in our samples was *B.tarsalis*, found in eight out of the eleven conservation units studied. Six species were found in only one unit each, namely *Buenoafemoralis*, *B.koina*, *B.platycnemis*, *B.pseudomutabilis*, *Enitharoidesbrasiliensis*, and *E.tricomerus* (Table 2), three of which are found in PNCD. Nevertheless, some are relatively widespread in the country, showing that more surveys are needed in order to better understand the distribution of backswimmers in northeastern Brazil. Five species, *B.amnigenus*, *B.platycnemis*, *B.tarsalis*, *Notonectadisturbata*, and *M.bentoi* were found outside conservation units in Alagoas and Sergipe states. All of them were also found inside conservation units in these and/or other states as well. The results achieved in this work greatly improve the knowledge on the distribution of Notonectidae in Brazil, filling a gap of biodiversity information for the northeastern states of the country.

**Table 2.** Distribution of Notonectidae species by their presence in Conservation Units sampled in this work. A.F.: Atlantic Forest; C. Caatinga; C.S. Cerrado Savannah.

State		AL					BA	CE	PE	PI	SE	
Conservation Unit		APACC	APAP	EEM	RESEX	RBPT	PNCD	EEA	PNCA	PNSC	PNSI	RBSI
Biome		A.F	A.F	A.F	A.F	A.F	C	C	C	C/C.S.	A.F/C	A.F
Subfamily	Species											
**Anisopinae**	*B.amnigenus*	–	–	–	–	–	**X**	**X**	**X**	**X**	–	–
	*B.femoralis*	–	–	–	–	–	–	–	–	**X**	–	–
	*B.fuscipennis*	–	–	–	–	–	–	**X**	**X**	–	–	–
	*B.koina*	–	–	–	–	–	**X**	–	–	–	–	–
	*B.konta*	**X**	–	**X**	–	–	**X**	**X**	–	–	**X**	–
	*B.mutabilis*	–	–	–	–	–	–	–	–	**X**	**X**	–
	*B.pallipes*	–	–	–	–	**X**	–	–	**X**	–	–	–
	*B.platycnemis*	–	–	–	–	–	–	–	**X**	–	–	–
	*B.pseudomutabilis*	–	–	–	–	–	**X**	–	–	–	–	–
	*B.salutis*	**X**	**X**	–	–	–	**X**	**X**	–	–	**X**	–
	*B.tarsalis*	**X**	**X**	–	–	**X**	**X**	**X**	**X**	**X**	–	**X**
	*B.unguis*	–	**X**	–	–	–	–	**X**	**X**	**X**	–	**X**
	*N.disturbata*	–	–	–	–	**X**	–	**X**	**X**	–	**X**	–
**Notonectinae**	*E.brasiliensis*	–	–	–	–	–	**X**	–	–	–	–	–
	*E.tricomerus*	–	–	**X**	–	–	–	–	–	–	–	–
	*M.bentoi*	**X**	–	**X**	–	–	**X**	**X**	**X**	–	**X**	–
	*M.brasiliensis*	–	–	–	**X**	–	–	**X**	–	–	–	–
	*M.membranacea*	–	–	–	–	–	**X**	–	–	–	–	**X**

## Supplementary Material

XML Treatment for Buenoa amnigenus

XML Treatment for Buenoa femoralis

XML Treatment for Buenoa fuscipennis

XML Treatment for Buenoa koina

XML Treatment for Buenoa konta

XML Treatment for Buenoa mutabilis

XML Treatment for Buenoa pallipes

XML Treatment for Buenoa platycnemis

XML Treatment for Buenoa pseudomutabilis

XML Treatment for Buenoa salutis

XML Treatment for Buenoa tarsalis

XML Treatment for Buenoa unguis

XML Treatment for Notonecta (Paranecta) disturbata

XML Treatment for Enitharoides brasiliensis

XML Treatment for Enitharoides tricomerus

XML Treatment for Martarega bentoi

XML Treatment for Martarega brasiliensis

XML Treatment for Martarega membranacea

## ﻿Additional information

### Author ORCIDs

Rafael Jordão https://orcid.org/0000-0002-0339-5293

Julianna Freires Barbosa https://orcid.org/0000-0002-5276-194X

Felipe Ferraz Figueiredo Moreira https://orcid.org/0000-0002-6692-0323

### Data availability

All of the data that support the findings of this study are available in the main text.
